# Tunable Dynamics via Dual‐Ion Modulation for Event‐based Data Processing Using a Highly Uniform and Self‐Rectifying Memristor Array

**DOI:** 10.1002/advs.75594

**Published:** 2026-05-10

**Authors:** Yoonho Cho, Dawon Kim, Jeonghong Lee, Dae‐won Kim, See‐On Park, Jongmin Bae, Taehwan Jang, Shinhyun Choi

**Affiliations:** ^1^ School of Electrical Engineering Korea Advanced Institute of Science and Technology (KAIST) Daejeon Republic of Korea; ^2^ Graduate School of Semiconductor Technology Korea Advanced Institute of Science and Technology (KAIST) Daejeon Republic of Korea; ^3^ Information and Electronics Research Institute Korea Advanced Institute of Science and Technology (KAIST) Daejeon Republic of Korea

**Keywords:** bio‐inspired computing, dual‐ion modulation, event‐based moving object classification, ion diffusion retarder, tunable dynamics

## Abstract

The demand for new computing architectures has greatly increased with the growth of data and the need for efficient data processing. Memristors are promising candidates for bio‐inspired computing hardware owing to their analog behaviors, simple structure, and simple fabrication process. Among them, interface‐type memristors have been extensively studied due to their high reliability and low power consumption. Despite these advantages, their spontaneous relaxation properties pose a challenge for bio‐inspired computing applications. In this article, we demonstrate the tunable dynamics of an interface‐type memristor through dual‐ion modulation by incorporating Ag nanoclusters (ion diffusion retarders), and successfully implement a 32 × 32 one‐resistor (1R) array (100% yield) with high temporal/spatial uniformity (σ/µ<3%) and high read rectifying ratio (>7 × 10^4^). The Ag nanoclusters combined with the oxygen anions modulate the conductance decay of the memristor by retarding the oxygen anion diffusion, enabling tunable dynamic behaviors. Leveraging these properties, we demonstrate a hardware implementation of the hierarchy of event‐based time surface algorithm (HOTS) using a highly uniform interface‐type memristor array. By demonstrating successful hardware implementation of exponential decaying kernels in the HOTS algorithm for the classification of moving objects (Poker Dynamic Vision Sensor), this work could pave the way toward resource‐efficient computing paradigms based on memristors.

## Graphical abstract

Tunable dynamics of an interface‐type memristor array enabled by dual‐ion modulation through Ag nanoclusters are demonstrated. A 32 × 32 one‐resistor (1R) array exhibits 100% yield with high temporal/spatial uniformity (<3%) and a rectification ratio of 10^5^. Based on the modulated decaying properties, the memristor successfully implements a hardware‐based moving object classification algorithm (HOTS) and achieves an accuracy of 96.5%.

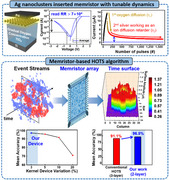

## 1 Introduction

The rise of artificial intelligence (AI) and machine learning (ML), further accelerated by the emergence of deep learning, has enabled the resolution of complex tasks in various fields, such as robotics, cognitive tasks, and the automotive industry [1]. It has been accompanied by the exponential growth in the amount of data to be processed and stored. In the conventional von Neumann architecture, the computation function and memory function are performed in each physically separated unit. As a result, it requires data transfer between the separated computing and memory units, which makes it unsuitable to handle these enormous amounts of data [2]. The elimination of data transfer between the computing and memory units offers significant advantages in terms of speed and low power consumption. As a result, bio‐inspired computing, which performs computation and memory functions in the same location, has gained great attention due to its potential to handle the increased amount of data.

Many emerging memory devices, such as Resistive Random Access Memory (ReRAM), Phase Change RAM (PCRAM), and Magnetoresistive RAM (MRAM), were widely studied to construct bio‐inspired computing architecture utilizing their inherent characteristics [3, 4, 5, 6, 7]. Among them, ReRAM, also called a memristor, has attracted many researchers as candidates for bio‐inspired computing hardware devices owing to its good analog memory characteristics, simple structure, and simple fabrication process. Moreover, the capability of vector‐matrix multiplication when fabricated in a crossbar array makes the memristor more promising as a device for constructing a bio‐inspired computing architecture [8, 9, 10]. Among various types of memristors, filamentary‐type memristors operate by changing their resistance state through the formation and rupture of conductive filaments. Generally, active metal cations such as Ag or Cu form conductive filaments in Conductive Bridge RAM (CBRAM), and the oxygen vacancies are the main sources forming a conductive filament in Valence Change Memory (VCM) [11, 12, 13, 14]. In constructing a successful bio‐inspired computing architecture, the spatial and temporal variations, and the overall yield of the memristor array are crucial to achieving high performance [15]. Despite their long retention and high on/off ratio, filamentary‐type memristors suffer from reliability issues due to the highly stochastic behavior of filament operation and the forming process [16, 17, 18, 19]. To address these issues, research on controlling the filament switching by confining the filament path utilizing the dislocation of the epitaxial layer or the porous structure has been conducted [20, 21]. These studies showed improved performance in spatial and temporal variations of the filamentary‐type memristors, but the issues regarding the inherent stochastic behavior of filament switching remained.

Studies on the interface‐type memristor (non‐filamentary type), where the resistance state of the memristor changes through the movement of oxygen anions, have been widely studied to mitigate the variation issues arising from the filament operation [22, 23, 24]. A previous study has demonstrated a great improvement in the reliability of the memristor by changing the resistance state of the device through the movement of oxygen anions throughout the entire bulk [25]. With a gradient oxygen concentration switching layer, the memristor showed its ability to demonstrate a highly uniform leaky‐integrate and fire (LIF) neuron model and sequential data computing. Nonetheless, a simple operating mechanism based on diffusion has limited the implementation of more complex functionalities in the device. The implementation of bio‐inspired computing architecture is feasible when utilizing devices that can store some of the previously stored information. Moreover, the ability to process multiple inputs and obtain outputs depending on the designated tasks is also necessary to emulate the computation mechanism of the biological brain.

The scalability of memristor arrays is also a critical consideration for applying them to practical applications. When memristors are integrated into a large crossbar array, undesired current paths, referred to as sneak currents, can degrade the array's performance since they affect the accurate programming of the selected cells. Integrating the memristor with the selector can be one solution to this problem, but it may lead to the degradation of the memristor performance and the reduced integration density. In this regard, architectural or algorithmic approaches have been proposed to mitigate the problems arising from the sneak current issues in a large passive crossbar array [26, 27]. Alternatively, another promising approach is to suppress sneak currents intrinsically at the device level by employing self‐rectifying memristors. Self‐rectifying memristors do not require any additional selectors, architectural or algorithmic strategies to mitigate the sneak current issues when integrated into a large‐scale array, thereby potentially reducing area overhead and improving scalability. A heterostructure or metal‐doped bilayer oxide layers were utilized to achieve self‐rectifying behavior and implement a passive crossbar array, and showed their performance in various security applications [28, 29, 30]. Also, a high rectification ratio was observed in HfO_x_‐based memristor through a rapid thermal annealing (RTA) process. RTA repairs a substantial number of oxygen vacancies in the HfO_x_ layer and suppresses the local conducting filament. Based on these properties, a self‐rectifying memristor array‐based real‐time autonomous driving system was successfully constructed and showed its ability to exhibit a robust attack resilience property [31].

In this study, we fabricated a highly uniform and self‐rectifying interface‐type memristor with Ag nanoclusters as ion diffusion retarders and demonstrated a reliable selector‐less one‐resistor (1R) array by achieving an 100% yield in a 32 × 32 array with low spatial variation. The self‐rectifying behavior was achieved by forming a large Schottky barrier at the interface of the electrode and the anodized TiO_x_ layer. Ag nanoclusters retard the spontaneous diffusion of oxygen anions by capturing the mobile oxygen, enabling the tunable dynamics of the memristor. The double exponential decaying property was also observed, and the mechanism of dual‐ion dynamics was studied through electrical and material analysis. Furthermore, multi‐level representation, which is an essential characteristic of implementing successful vector‐matrix multiplication, was also demonstrated. In addition, exponential decaying kernels of the hierarchy of event‐based time surface algorithm (HOTS) were demonstrated by utilizing the double exponential decaying properties of the memristor. The generation of time surfaces using a 7 × 7 memristor array was experimentally demonstrated, and the highly uniform characteristics of a hardware‐implemented exponential decaying kernel showed its advantage in extracting accurate spatiotemporal features for resource‐efficient event‐based computing algorithms. These results highlight the potential of the memristor with Ag nanoclusters as hardware devices for constructing a bio‐inspired computing architecture.

## 2 Results and Discussion

### 2.1 Highly Uniform and Self‐Rectifying Interface‐type Memristors with Ag Nanoclusters

**FIGURE 1:**
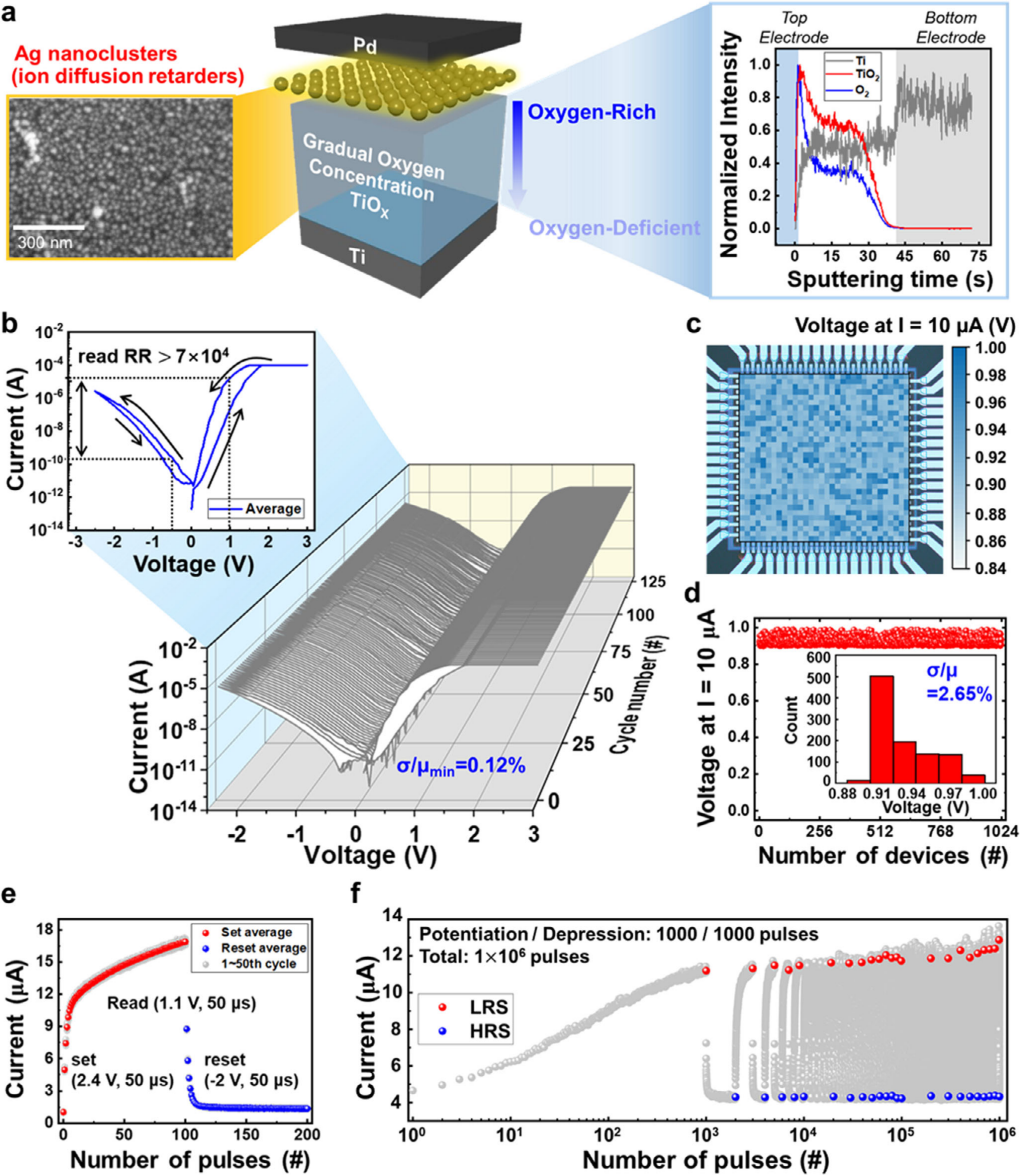
**Highly uniform and self‐rectifying interface‐type memristor with Ag nanoclusters**. (a) Schematic of interface‐type memristor with Ag nanoclusters. The SEM image of Ag nanoclusters deposited on top of the anodized TiO_x_ layer is shown inside the orange box, and the results of the TOF‐SIMS depth profile are shown inside the blue box, (b) Consecutive 125 DC cycles in the *I*–*V* curve. A minimum temporal variation of 0.12% and a large read rectifying ratio over 7 × 10^4^ is observed, (c) A 2D heat map showing 100% yield of the 32 × 32 array. Each point represents the voltage when the device reaches 10 µA in LRS during the DC sweep, (d) Low spatial variation (2.65%) of all 1024 devices in the array calculated based on the voltage obtained in (c), (e). Conductance update characteristics in 50 consecutive cycles. 100 set (2.4 V, 50 µs) and 100 reset pulses (−2 V, 50 µs) were applied at each cycle. The read pulses (1.1 V, 50 µs) were applied after each set and reset operation. The red curve and the blue curve indicate the average response of the set and reset stages during 50 cycles, respectively, (f) Endurance characteristic of the memristor during the entire cycling range.

Figure 1a illustrates the schematic of a highly uniform and self‐rectifying interface‐type memristor with Ag nanoclusters (ion diffusion retarders). As shown in the image of the scanning electron microscope (SEM), Ag nanoclusters are uniformly distributed on the top of the TiO_x_ switching layer. A TiO_x_ switching layer of the device was fabricated by oxidizing a thin Ti film through an anodization process (the detailed information for the fabrication is described in the Experimental section). Since the anodization is a top‐down process, the top region of Ti undergoes a longer oxidation time compared to the bottom region, resulting in the upper part of the anodized layer exhibiting a higher oxygen concentration than the lower part. Consequently, a TiO_x_ switching layer with a gradual oxygen concentration is formed. The results of the time of flight secondary ion mass spectrometry (TOF‐SIMS) in Figure 1a illustrate the gradual oxygen concentration in the anodized TiO_x_ layer. The concentrations of TiO_2_ and O_2_ are highest at the top region of the anodized layer (Pd/TiO_x_ interface), and they gradually decrease as they reach the bottom region (TiO_x_/Ti interface). Furthermore, the lowered activation energy of oxygen anions during anodization allows the movement of oxygen anions throughout the entire bulk during the operation [32]. This bulk resistive switching mechanism of the device allows it to show a high on/off ratio.

Figure 1b shows the typical *I*–*V* curves of the interface‐type memristor with Ag nanoclusters during consecutive 125 DC cycles, where a maximum on/off ratio over 10^3^ and a large read rectifying ratio (RR>7 × 10^4^) are observed. The read RR of the device was calculated as the ratio of the current at positive read voltage to that of negative half read voltage (I_Vread_/I_‐Vread/2_) based on the half‐voltage scheme. High set RR (>2 × 10^4^) is also observed in Figure S1, which is calculated as the ratio of the current at positive set voltage to that of negative half set voltage (I_Vset_/I_‐Vset/2_). The high read RR originates from the Schottky barrier between the top electrode and the TiO_x_ switching layer. Selecting a top electrode with a high work function, such as Pd (φ = 5.12 eV [35]), leads to self‐rectifying behavior with a read RR larger than 6 × 10^6^, as a large Schottky barrier is formed at the interface of the switching layer and the electrode (Figure S2a,b). The high Schottky barrier is preserved even after inserting Ag nanoclusters, since the discrete nature of Ag nanoclusters ensures that Pd continues to govern the contact energetics. Indeed, the Ag nanoclusters affect the rectification ratio of the device. As shown in Figure S2c,d, a significant degradation of read RR to 200 is observed when a complete Ag film forms a contact with a TiO_x_ layer, since the low work function of Ag (φ = 4.26 eV [33]) results in the formation of a low Schottky barrier at the interface of Ag/TiO_x_ layer. However, for discretely distributed Ag nanoclusters, the contact energies are not solely governed by Ag itself. It allows the Pd interface to continue playing a dominant role in determining the Schottky barrier. As a result, although the read RR is reduced compared to the device without Ag nanoclusters, it remains a high read RR (>7 × 10^4^), preserving self‐rectifying behavior. As illustrated in Figure S3, this self‐rectifying behavior poses a significant advantage when implemented in a large crossbar array since it does not require integration with a selector device [15, 34, 35]. Leveraging this property, we have successfully demonstrated a 32 × 32 1R array with 100% yield (Figure 1c).

In addition, we performed consecutive 125 DC cycles on 15 different devices within the array to analyze the temporal variation (Figure S4). The temporal variation was calculated by measuring the voltage when the device reached 10 µA in the low‐resistance state (LRS). A highly uniform characteristic is observed in the *I‐* *V* curves, with a minimum and maximum temporal variation (σ/µ) of 0.12% and 1.67%, respectively, over 125 DC cycles (Figure S5a,b). Furthermore, the spatial variation of all 1024 devices in our 32 × 32 array was analyzed. As shown in Figure 1d, a low spatial variation of 2.65% further confirms the highly uniform characteristics of the memristor with Ag nanoclusters. We also investigated the conductance update characteristics to examine the pulse response of the memristor. 100 set (2.4 V, 50 µs) and 100 reset pulses (−2 V, 50 µs) were applied at each cycle. The read pulses (1.1 V, 50 µs) were applied after each set and reset operation. The device shows low temporal variation of 1.16% and 2.01% in the set and reset stages over 50 consecutive cycles, respectively, further confirming its highly uniform characteristics under pulse operation (Figure 1e). The size dependency is studied to examine the non‐filamentary operation mechanism of the device. The memristor does not show size dependency when it operates through a locally formed filament, since most of the current flows through the conductive filament. However, the difference in the current of the memristor with various feature sizes exists when the device operates through the movement of ions through the entire bulk. The results shown in Figure S6 demonstrate that the device operates by the movement of oxygen anions throughout the entire bulk as the current of the device increases with the cell size. The endurance characteristic of the device is also investigated by applying 500 consecutive identical pulse schemes. Each cycle consisted of 1000 set (3.2 V, 50 µs) and 1000 reset (−2.2 V, 50 µs) pulses, and the read pulses (1.1 V, 50 µs) that are applied after each set and reset pulse. The result in Figure 1f demonstrates that the device exhibits reliable switching characteristics without severe degradation during 1 × 10^6^ consecutive pulses. These highly uniform and reliable characteristics in both DC and pulse operating conditions originate from the non‐filamentary switching mechanism and the absence of the forming process [18, 19, 36]. The forming process influences the electrical characteristics of the device as it involves the soft breakdown process of the device. The forming‐free nature of the proposed device allows it to exhibit both low temporal and spatial variations while maintaining a high endurance of up to 10^6^ cycles.

### 2.2 Effect of Ag Nanoclusters on the Decaying Properties

**FIGURE 2:**
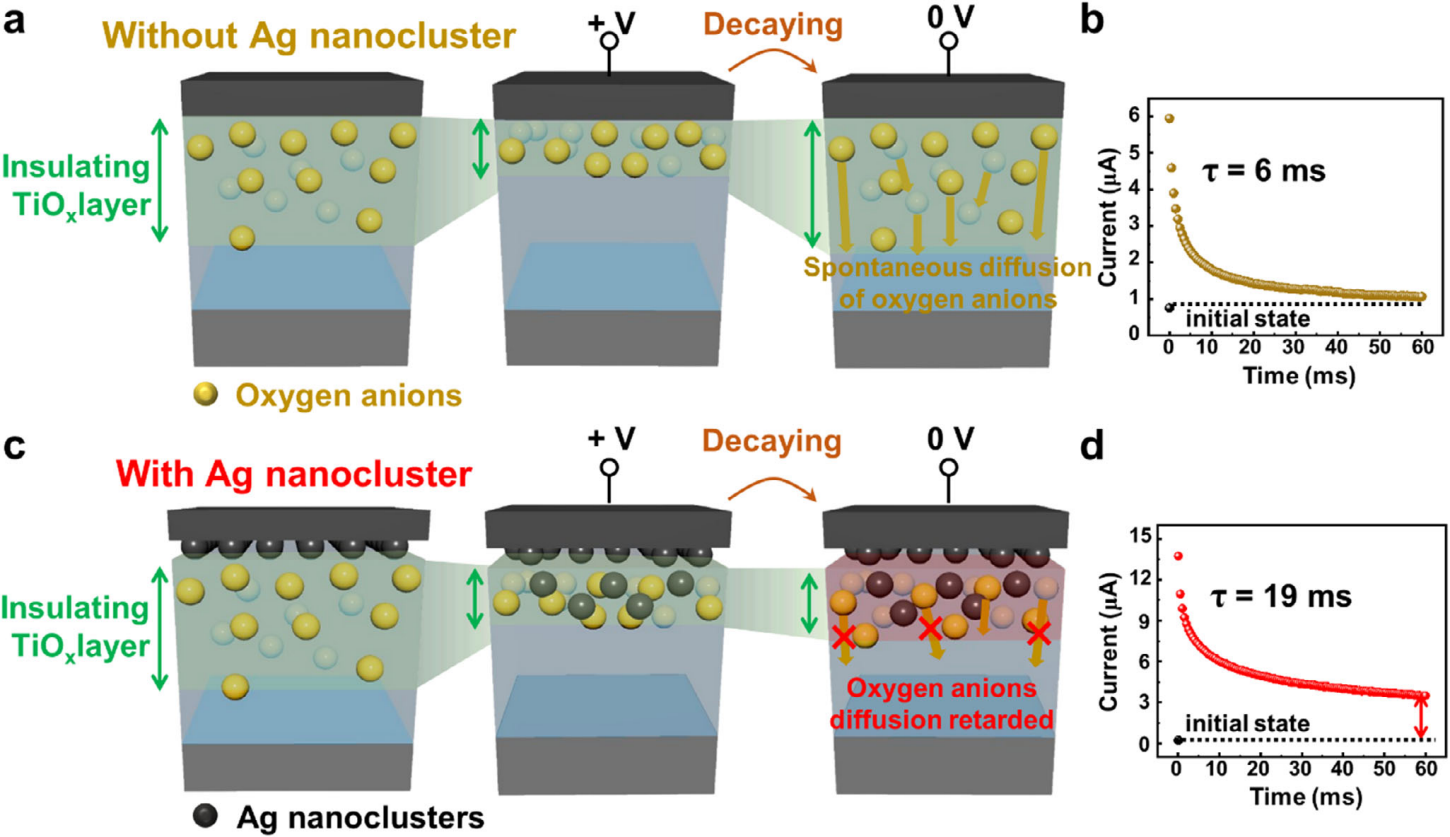
**Operation schematic of interface‐type memristors**. (a–d) Schematic describing the operation mechanism before the operation, when the positive bias is applied to the top electrode, and after the bias is removed in the interface‐type memristors and corresponding time constant results. (a,b) without Ag nanoclusters, (c,d) with Ag nanoclusters.

Figure 2 elucidates the effect of Ag nanoclusters and their role as ion diffusion retarders by comparing the operation behavior of the device without and with Ag nanoclusters. When a positive bias is applied to the device, as shown in Figure 2a, oxygen anions migrate to the upper region of the anodized layer, decreasing the thickness of the insulator‐like TiO_x_ layer. The device then turns into LRS. In a device without Ag, the thickness of the insulator‐like TiO_x_ layer returns to the thickness before potentiation after the bias is removed, as the oxygen anions spontaneously diffuse back to their original state. This results in the device changing its state into a high resistance state (HRS), showing its fast decaying property. As shown in Figure 2b, the memristor without Ag nanoclusters exhibits fast decaying properties with a time constant of 6 ms, where the time constant is defined as the time at which the current of the memristor decays to 1/*e* of its initial value. The device with Ag nanoclusters shows identical operation behavior during the potentiation period, but a difference exists in the decaying process and the current level. Compared to the device without Ag nanoclusters, the diffusion of oxygen anions is retarded when the bias is removed because some of the diffusible oxygen anions are captured at the interface of Ag nanoclusters and the anodized TiO_x_ layer, as illustrated in Figure 2c. The retardation of oxygen anions induced by Ag nanoclusters modulates the transition speed of the thickness of an insulator‐like TiO_x_ layer. As a result, the increase in the time constant from 6 to 19 ms is observed, demonstrating the tunable dynamics through dual‐ion modulations. As shown in Figure 2d, the device still retained some of its memory even 60 ms after potentiation, indicating its ability to represent temporal information of the input through tunable dynamics. Also, the difference in the electrical conduction mechanism resulting from the insertion of Ag nanoclusters affects the current level. When Pd (high work function metal) is used as the top electrode, electrons have to overcome the barrier under a small positive bias (Figure S7a,b). In contrast, the reduced band bending, when Ag (low work function metal) is used as the top electrode, facilitates the flow of electron transport from the bottom electrode to the top electrode (Figure S7c,d). When Ag nanoclusters are discretely distributed at the Pd/TiO_x_ interface, the combined effect of both Pd/TiO_x_ and Ag/TiO_x_ cases contributes to the overall conduction of the memristor. As a result, a slight increase in the current level is observed in the device with Ag nanoclusters (Figure S8).

**FIGURE 3:**
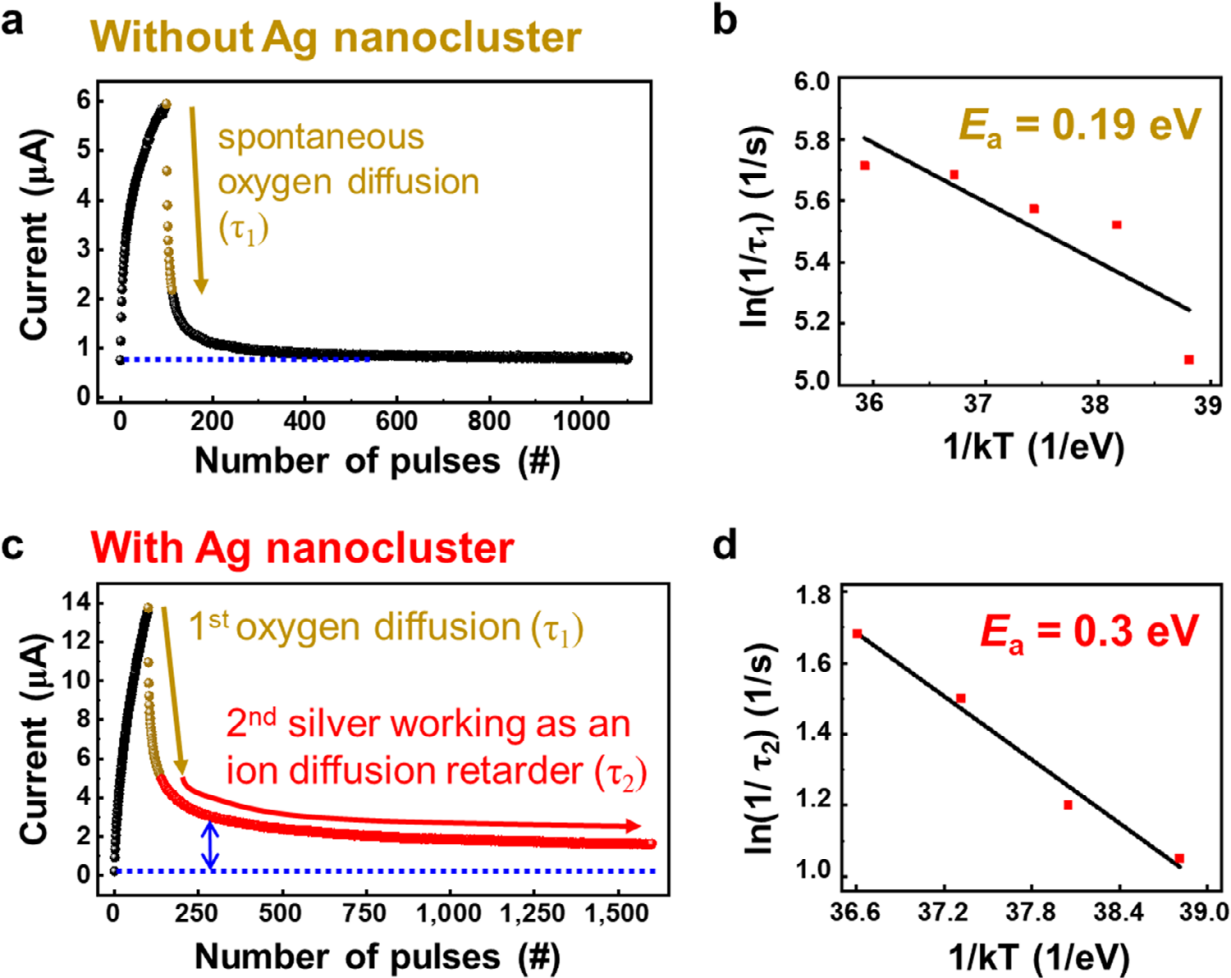
**Electrical analysis of the decaying process of the memristors**. (a) Decaying process of the memristor without Ag nanoclusters. 100 set pulses (3.6 V, 80 µs) were applied and subsequently, 1000 read pulses (1.4 V, 100 µs) without the reset pulses were applied to monitor the conductance decay of the memristor, (b) Activation energy calculated from the time constants at various temperatures, (c) Decaying process of the memristor with Ag nanoclusters. τ_1_ represents the time constant of the first decaying process of the memristor and τ_2_ represents the time constant of the second decaying process of the memristor. 100 set pulses were applied (2.8 V, 80 µs) and subsequently, 1500 read pulses (1.1 V, 100 µs) without the reset pulses were applied to monitor the decaying process of the memristor, (d) Activation energy calculated from the time constants of the second exponential term at various temperatures.

To compare the operation behavior between the memristor without and with Ag nanoclusters, the decaying process of both devices was monitored. Figure 3a illustrates the decaying process of the interface‐type memristor without Ag nanoclusters. In this case, the movement of oxygen anions in response to the applied bias mainly affects the resistance state of the device. During the potentiation period, oxygen anions drift toward the top electrode under the applied positive bias, creating a non‐equilibrium oxygen distribution within the TiO_x_ layer. Once the bias is removed, the resulting concentration gradient provides a thermodynamic driving force for diffusion. Therefore, the device shows single exponential decaying characteristics as the oxygen anions gradually redistribute toward their equilibrium state, resulting in the fast self‐decaying property. The movement of oxygen anions across the whole bulk and its effect on the resistance state of the device was demonstrated by calculating the activation energy (*E*_a_). As described in Figure 3b, the activation energy calculated from the Arrhenius plot is about 0.19 eV, which aligns well with the activation energy of oxygen anions in the anodized TiO_x_ layer [25]. On the other hand, the decaying process of the interface‐type memristor with Ag nanoclusters, as shown in Figure 3c, can be described by double exponential decaying functions due to the role of Ag nanoclusters as oxygen anion diffusion retarders, as follows [37]:

(1) $$I(t)=I_{0}+A_{1}e^{(-t/\tau_{1})}+A_{2}e^{(-t/\tau_{2})}$$

In the above function, *I*_0_ is the offset current, and *A*_1_ and *A*_2_ are the constants. The time constant of the first exponential term (τ_1_) represents the first decaying process, which is dominated by the diffusion of oxygen anions, similar to that of the memristor without Ag nanoclusters. The time constant of the second exponential term (τ_2_) represents the second decaying process of the memristor, where Ag nanoclusters retard the diffusion of oxygen anions [37]. During memristor operation, Ag nanoclusters affect the resistance state of the memristor as well as the oxygen anions. Oxygen can be dissociated at the surface of Ag nanoclusters, and subsequent oxidation can take place through the adsorbed oxygen [38]. These processes limit the number of mobile oxygen anions responsible for diffusion, thereby retarding their movement. As a consequence, the self‐decaying process of the memristor is modulated, retaining a partial memory of previous inputs even in the absence of external bias, as illustrated in Figure 3c.

To investigate the role of Ag nanoclusters as ion diffusion retarders, the time constants of the first (τ_1_) and second exponential term (τ_2_) were measured at various temperatures to calculate the activation energy. The activation energy of the first exponential term is 0.19 eV, identical to that of the memristor without Ag nanoclusters, implying that the device still operates by the movement of oxygen anions in the anodized TiO_x_ layer (Figure S9). As shown in Figure 3d, the calculated activation energy of the second exponential term is about 0.3 eV, which is similar to the activation energy of silver oxidation by atomic oxygen [39]. These results demonstrate that Ag nanoclusters induce the tunable dynamics of the memristor by impeding the diffusion of oxygen anions. To further examine the effect of Ag content on the device performance, devices with different amounts of Ag (nominal thicknesses 1, 2, and 3 nm) were compared. The SEM image reveals that as the amount of Ag increased, the average radius of Ag nanoclusters enlarged from approximately 14.1 to 15.7 nm and 28.1 nm for the 1, 2, and 3 nm cases, respectively. However, the overall surface coverage (%) remained within a similar range of 43%–45% (Figure S10). The *I*–*V* curves of the memristor with different amounts of Ag nanoclusters are shown in Figure S11. Almost identical read RR of 2 × 10^5^ and 1 × 10^5^ are observed for the devices with 43.4% and 43.1% surface coverage, respectively (Figure S11a,b). A slightly lower read RR (1 × 10^4^) is observed for the device with 45.1% surface coverage (Figure S11c). A lowered read RR is attributed to the increase in the Ag surface coverage, resulting in the increased effect of Ag in the recitification mechanism. However, it still shows a high read RR, and the read RR values of the three different cases were comparable. The on/off ratio was also analyzed by calculating the current ratio between the HRS and LRS at 1 V in the *I*–*V* curve. As illustrated in Figure S12, the on/off ratios of the devices with 43.4%, 43.1%, and 45.1% surface coverage are 104, 138, and 83, respectively, indicating that the on/off ratios are also comparable across the three devices, similar to the trend shown in the read RR. Moreover, the decaying properties of the memristor showed no big difference with varying amounts of Ag nanoclusters, exhibiting similar decaying properties and activation energy in the second exponential term (Figure S13). These results suggest that the surface morphology, particularly whether it exists as discrete nanoclusters or as a continuous film, primarily governs the electrical performance, such as RR, on/off ratio, and most importantly, the tunable dynamics of the device. This trend is also observed in the normalized current results, as shown in Figure S13d. Devices with different amounts of Ag nanoclusters do not show distinguishable differences in the decaying characteristics, while the device without Ag nanoclusters shows fast decaying properties due to the spontaneous diffusion of oxygen anions.

**FIGURE 4:**
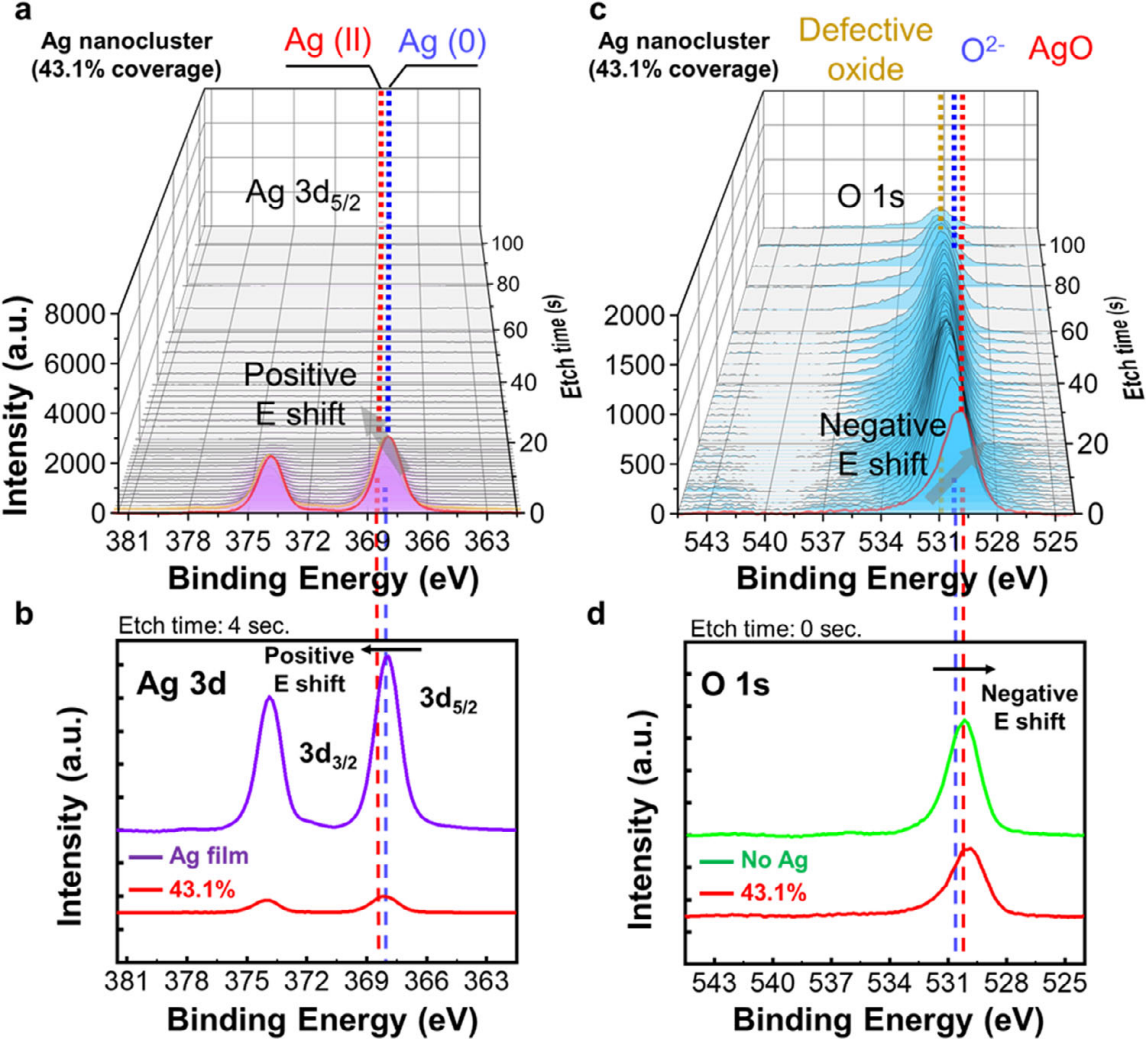
**XPS depth profile analysis of the memristor with Ag nanoclusters**. (a) Ag 3d orbital XPS depth profile results of the interface‐type memristor with Ag nanoclusters showing 43.1% surface coverage, (b) Ag 3d orbitals XPS spectra of the memristor with Ag nanoclusters (red curve) and Ag film (purple curve) at 4 sec. etch time, (c) O 1s orbitals XPS depth profile results of the interface‐type memristor with Ag nanoclusters showing 43.1% surface coverage, (d) O 1s orbitals XPS spectra of the memristor with Ag nanoclusters (red curve) and without Ag nanoclusters (green curve) at 0 sec. etch time.

Additionally, the X‐ray photoelectron spectroscopy (XPS) depth profile analysis was conducted to identify the mechanisms of Ag nanoclusters acting as ion diffusion retarders. As shown in the XPS depth profile results of thick Ag film on top of the anodized TiO_x_ layer, Ag 3d_5/2_ spectra of metal Ag are observed at 367.9 eV, as shown in Figure S14a. The XPS depth profile results of the device with Ag nanoclusters (43.1% surface coverage) are shown in Figure 4a. Compared to the Ag 3d_5/2_ spectra with a peak at 367.9 eV in the complete film, a positive peak shift of Ag 3d_5/2_ spectra is observed in Figure 4b, exhibiting a peak at 368.2 eV [40, 41]. The positive peak shift of Ag 3d_5/2_ spectra can be explained by the formation of AgO_y_ at the interface of Ag nanoclusters and the anodized TiO_x_ layer, which is formed by the oxidation of Ag nanoclusters with oxygen anions [42]. To demonstrate the formation of AgO_y_, the analysis of XPS depth profile results of O 1s orbitals for the device with Ag nanoclusters was also conducted (Figure 4c). The peak of the O 1s spectra is observed at 530.2 eV at the surface of the anodized TiO_x_ layer in a device without Ag nanoclusters (Figure S15), which corresponds to the O 1s peak of oxygen‐rich TiO_2_ [43]. With the addition of Ag nanoclusters on top of the anodized TiO_x_ layer, a negative shift from 530.2 to 529.8 eV is observed, as illustrated in Figure 4d. The negative shift of the O 1s peak with the positive shift of the Ag 3d_5/2_ peak indicates the formation of AgO_y_ at the interface of Ag nanoclusters and the TiO_x_ layer, which plays a role in retarding the diffusion of oxygen anions by capturing the mobile oxygen anions responsible for diffusion. The analysis of the XPS depth profile was also conducted for Ag nanoclusters with different surface coverage (Figure S14b–d). Similar to the device with 43.1% surface coverage, Ag 3d_5/2_ spectra show positive peak shifts from 367.9 to 368.2 eV and 368.5 eV for the devices with 43.4% and 45.1% surface coverage, respectively (Figure S16a). Moreover, negative peak shifts at O 1s spectra from 530.2 to 529.8 eV are also observed in both devices with 43.4% and 45.1% surface coverage, as depicted in Figure S16b. These results prove the formation of AgO_y_ by the oxidation of Ag nanoclusters, and the analogous peak shifts observed in devices with different surface coverage demonstrate the similarly tunable dynamics of the memristors.

**FIGURE 5:**
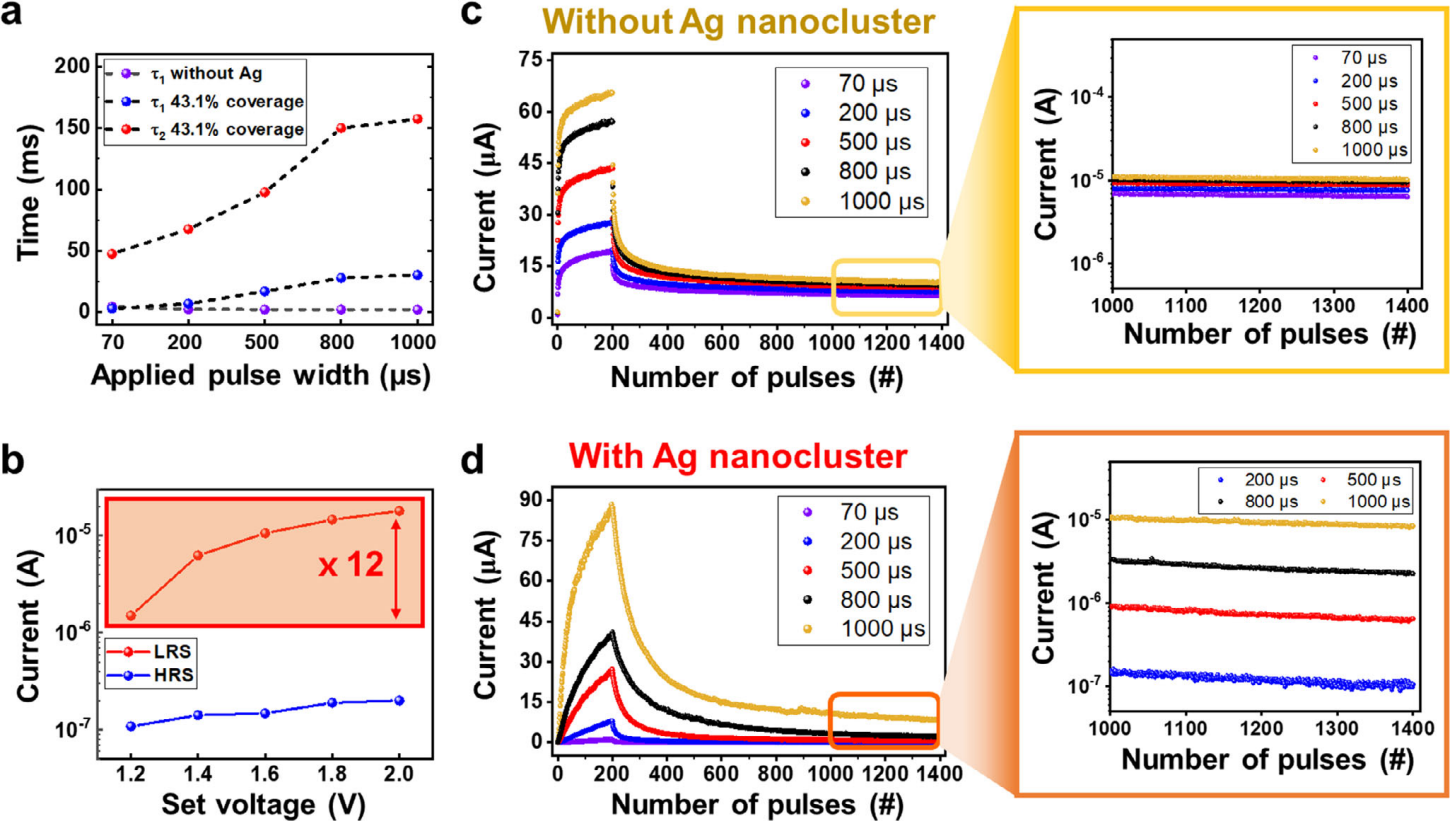
**Tunable dynamics of the interface‐type memristor with Ag nanoclusters**. (a) The change in the time constant of the first and second exponential terms in the device without and with Ag nanoclusters according to the applied pulse width. The device with Ag nanoclusters shows a higher dynamic range of time constant compared to the device without Ag nanoclusters, (b) The various conductance states of the memristor with Ag nanoclusters obtained by varying the set voltage during the DC sweep, (c,d) The decaying curve of the memristors without and with Ag nanoclusters with different pulse widths. The extended graph inside each box describes the steady‐state region of the device, (c) No difference in the current levels is observed with the change in the applied pulse width, (d) Distinguishable differences in the current levels are observed with the change in the applied pulse width.

Figure 5a illustrates the dynamic range of the time constants for the first (τ_1_) and second (τ_2_) exponential terms with the change in the applied pulse width in the device without and with Ag nanoclusters. In a device without Ag nanoclusters, τ_1_ remains relatively unchanged across varying pulse widths. However, the device with Ag nanoclusters exhibits an increase in τ_1_ and a more pronounced dynamic range in τ_2_ with increasing pulse widths, demonstrating its tunable dynamics. Depending on the applied pulse width, the amount of Ag nanoclusters inserted inside the TiO_x_ layer varies, and these differences affect the number of oxygen anions diffusing back to their original states. Therefore, a more prominent change in the decaying properties can be observed in the device with Ag nanoclusters. The device's potential to represent various conductance states was also demonstrated by varying the set voltage, as elucidated in Figure 5b. The currents of HRS and LRS were obtained at 1 V during the DC sweep. The change in set voltage affects the LRS, as the amount of oxygen anions moving across the entire bulk varies with the applied voltage. Higher set voltages raise more oxygen anions to the upper region, decreasing the thickness of the insulator‐like TiO_x_ region. A maximum difference in the LRS of up to 12 times can be observed depending on the set voltage, and this inherent switching mechanism of the device enables the device to represent analog LRS behavior by varying the set voltage. The comparison of the steady‐state current of the device without and with Ag nanoclusters was also conducted. After applying 200 set pulses (3.2 V) with different pulse widths ranging from 70 µs to 1 ms, the steady‐state current was measured by applying the read pulses (1.4 V, 70 µs). In the device without Ag nanoclusters, the bulk movement of oxygen anions is the dominant factor influencing the device's resistance change. Therefore, the maximum current is affected by the applied pulse width since the number of oxygen anions migrating to the region near the top electrode varies. However, these anions spontaneously diffuse back to their original state regardless of the applied pulse width in the steady state. As a result, no distinguishable difference in the steady‐state current is observed (Figure 5c). On the other hand, noticeable differences in the steady‐state current are observed in the device with Ag nanoclusters, as shown in Figure 5d. When the positive bias is applied to the top electrode, Ag nanoclusters move toward the bottom electrode. Since the electrons are sufficiently provided from the bottom Ti electrode due to the low Schottky barrier between the Ti and the anodized TiO_x_ layer, Ag nanoclusters inserted inside the TiO_x_ layer redistribute according to the applied pulse width. These redistributions of Ag nanoclusters modulate the hopping current in the switching layer, which induces the resistance changes of the device and allows the realization of multiple resistance states depending on the applied pulse width. These results demonstrate the potential of the device with Ag nanoclusters to exhibit multi‐level representation in the steady‐state region.

### 2.3 Hardware Implementation of HOTS Algorithm

HOTS is a moving object classification algorithm that utilizes spatiotemporal features called time surfaces, obtained from asynchronously acquired visual scenes [44]. In conventional frame‐based vision systems, all information from the moving object over time is utilized to acquire its feature representation and synchronously process data through the fixed time units or clocks. Then, the extraction of feature representation is achieved by fine‐tuning the weights across multiple layers composed of basic feature extraction steps, including spatial convolution, pointwise operations, and pooling mechanisms [45, 46, 47]. However, these frame‐based vision systems suffer from issues including motion blur when capturing the rapid movement of objects, and are susceptible to noise under low‐light conditions. Motivated by these challenges, event‐based vision systems inspired by biological neural systems have attracted considerable attention, as they asynchronously process only change‐driven events from the surrounding environments. Since the asynchronous event streams are typically sparse in terms of spatiotemporal aspects, computational costs can be reduced by minimizing data redundancy and preventing unnecessary data processing.

**FIGURE 6:**
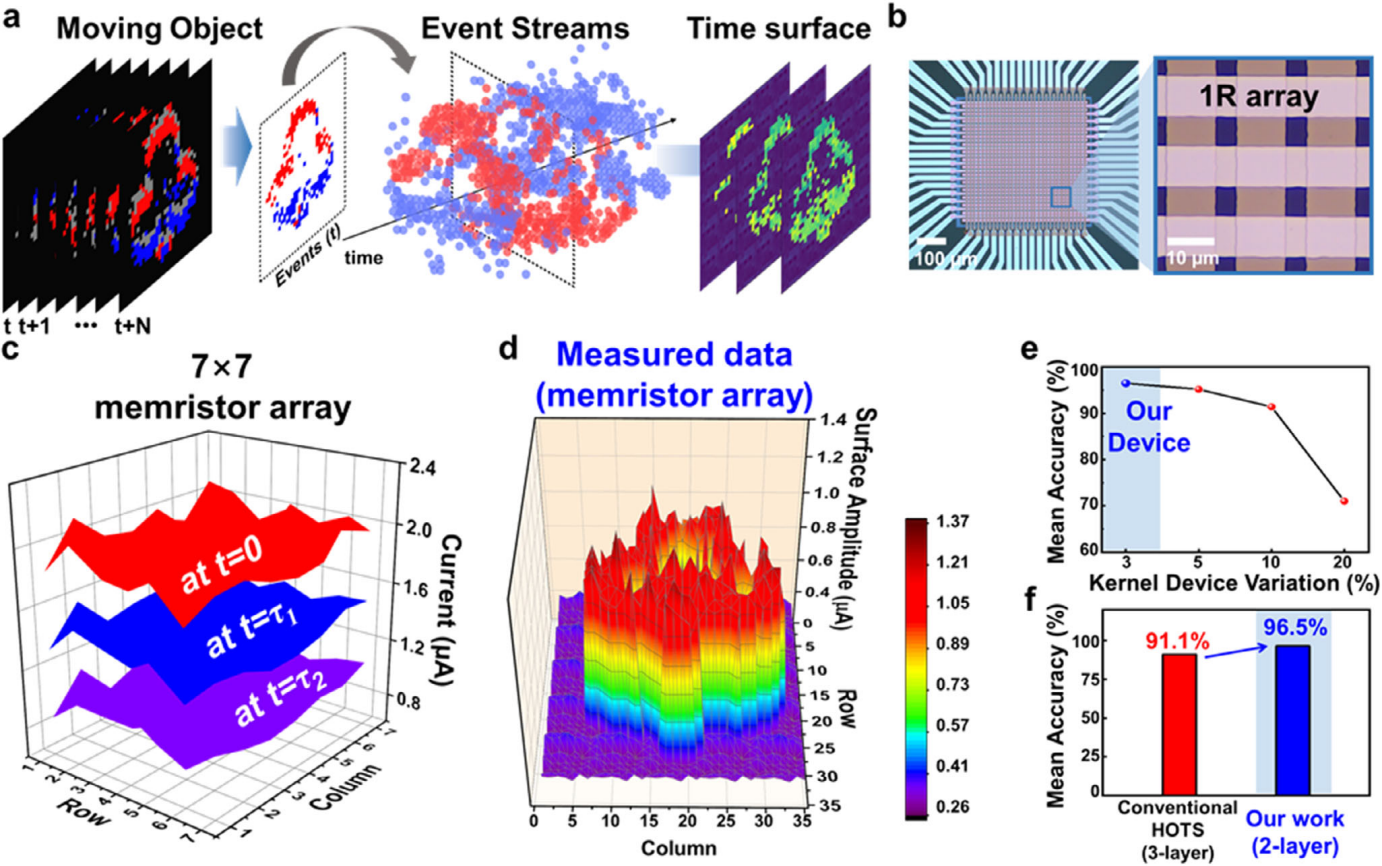
**Event‐based moving object classification algorithm and its hardware implementation using the memristor array with Ag nanoclusters**. (a) Schematics of capturing the events in the moving object and the generation of event streams. The blue dot indicates the decrease in the light intensity of the pixel, and the red dot indicates the increase in the light intensity of the pixel. Then, the time surfaces are generated by applying the exponential decaying kernel to the event streams, (b) Optical image of 32 × 32 selector‐less 1R memristor array, (c) 3D representation of the highly uniform conductance states of the memristor array at t = 0, t = τ_1_, and t = τ_2,_ (d) Time surfaces generated by using memristor array as exponential decaying kernels, (e) Mean classification accuracy as a function of kernel device variation, (f) Histogram representing the performance between the conventional HOTS architecture with a single exponential decaying kernel (red) and our HOTS architecture using double exponential decaying kernel (blue).

Figure 6a illustrates the schematic of generating time surfaces using a highly uniform interface‐type memristor with Ag nanoclusters in the HOTS algorithm. As the object changes its position over time, the events occur in the pixels that experience the light intensity change. These changes produce sparse and asynchronous event streams, which are then convolved with an exponential decaying kernel at the pixels and their surrounding neighbors to construct time surfaces. The resulting time surfaces provide dynamic spatiotemporal features since the exponential decaying kernel integrates the information of previously occurred events. After training time surfaces through an online clustering method, these time surfaces serve as features during the classification phase. When implementing the exponential decaying kernel with hardware devices, the uniformity of the kernel devices is an important factor to consider. Variations among kernel devices strongly affect the consistency of time surface formation during training and classification, with poor uniformity ultimately degrading the classification accuracy [48]. Moreover, a single exponential decaying kernel is used in the conventional HOTS architecture [44, 49]. To integrate spatiotemporal features over multiple time scales, multiple layers with progressively increasing time constants are employed (Figure S17). However, when the kernels exhibit a double exponential decaying property, the HOTS architecture can be realized with fewer layers, as every single layer can capture both short‐ and long‐term temporal dynamics [50].

As shown in Figure 6b, the memristor with Ag nanoclusters can be integrated into a selector‐less 1R array with a size of 32 × 32, owing to its self‐rectifying property. In the design of an exponential decaying kernel using the memristor array, a 7 × 7 sub‐array was used. The proposed memristor array is well‐suited for hardware implementation of the kernel due to its highly uniform characteristics across all 49 measured devices. Highly uniform conductance states of the memristor array measured at t = 0, t = τ_1_, and t = τ_2_ are illustrated in Figure 6c. Moreover, the spatial variation for the time constant in the first (τ_1_) and second (τ_2_) exponential terms was 3.64% and 2.39%, respectively, further verifying its reliable characteristics (Figure S18). To investigate the effect of kernel device variations on learning spatiotemporal features from event‐based inputs, time surfaces generated by memristors were compared to those produced under ideal kernel conditions, in which the spatial variation of the kernel was assumed to be zero (Figure S19). As illustrated in Figure 6d, time surfaces obtained using the memristors closely resemble those generated under ideal conditions, which results from highly uniform characteristics of the memristor array. Subsequently, our two‐layer HOTS architecture incorporating the double exponential decaying property was applied to the Poker Dynamic Vision Sensor (DVS) classification. The results in Figure 6e show that the highest mean accuracy of 96.5% was achieved when the time surfaces were generated using the memristors. When the variation among the kernel devices increased to 5% and 10%, the classification accuracy decreased to 95.2% and 91.4%, respectively. Further degradation was observed at 20% variation, with the mean accuracy dropping to 71%. The degradation of classification performance arises from non‐uniformity among kernel devices, causing identical event magnitudes to be integrated differently depending on their pixel locations. As the exponential decay kernel inherently encodes spatiotemporal information, significant device‐to‐device variation can compromise temporal integration accuracy, leading to spatial misinterpretation and reduced fidelity in the learned spatiotemporal features. Furthermore, the performance of the proposed HOTS model was compared with the conventional HOTS model, which employs a single exponential decaying kernel in the time surface generation. As shown in Figure 6f, our two‐layer HOTS architecture, leveraging the double exponential decaying behavior of the memristor array, outperformed the conventional three‐layer HOTS architecture, achieving a mean classification accuracy of 96.5% compared to 91.1% for the latter. The improved performance of our proposed model, despite using fewer layers, is attributed to the ability to capture features across multiple temporal scales from the event streams. These results demonstrate that the proposed interface‐type memristor array with Ag nanoclusters can serve as building blocks for successful hardware implementation of bio‐inspired computing systems.

## 3 Conclusion

The simple dynamics of the interface‐type memristors have made it difficult to utilize them in constructing bio‐inspired computing architecture, despite their improved reliability and low power consumption. In this study, we have successfully demonstrated tunable dynamics of a gradual oxygen concentration TiO_x_ layer‐based interface‐type memristor through inserting Ag nanoclusters, which shows a high on/off ratio over three orders of magnitude and a high rectifying ratio over four orders of magnitude. A 32 × 32 array with 100% yield has been achieved, and the array also exhibits low temporal and spatial variation due to its non‐filamentary bulk operation mechanism and the absence of a forming process. The tunable dynamics of the memristor are achieved through dual‐ion modulation, where Ag nanoclusters effectively retard the diffusion of oxygen anions. The role of Ag nanoclusters has been investigated by calculating the activation energy from the time constants obtained from various temperatures and XPS depth profile analyses. The activation energy results and the analysis of the XPS depth profile demonstrate that the oxidation of Ag nanoclusters affects the number of diffusible oxygen anions, thereby modulating the decaying characteristics of the device. Leveraging the highly uniform and reliable characteristics with its double exponential decaying properties, we have demonstrated the hardware implementation of an event‐based moving object classification algorithm using the proposed memristor array. The high classification accuracy is achieved when using our memristor array as an exponential decaying kernel, serving as a cornerstone for constructing a successful bio‐inspired computing architecture based on memristors.

## 4 Experimental Section

### 4.1 Device Fabrication

The device was fabricated on a silicon wafer with 100 nm of dry oxidized SiO_2_. First, a Ti (50 nm) bottom electrode with various feature sizes was patterned by photo‐litography and deposited by an electron‐beam evaporator, followed by a lift‐off process to define the bottom electrode. Next, a thin Ti film (12 nm) for the anodization was deposited globally on the whole wafer by an electron‐beam evaporator. Subsequently, anodization was performed in an electrolyte composed of a total of 0.1 m NaOH and NH_4_F in H_2_O. During the anodization, 10 V of constant voltage was applied to the sample for 1 min [51, 52, 53]. The anodization of 12 nm Ti film results in the formation of 35 nm anodized TiO_x_ layer. Then, dry etching by a Reactive Ion Etching (RIE) is performed to remove the unnecessary switching layer formed on top of the bottom electrode using PR (Photo‐Resist) masks. Finally, Pd(50 nm)/Ag top electrode was patterned by lithography and deposited by an electron‐beam evaporator with the lift‐off process.

### 4.2 Electrical Measurements

All DC measurements were performed using a parameter analyzer (Keithley 4200A‐SCS) with a conventional probe station. Pulse measurements were performed using a pulse measurement unit (PMU), with current responses measured by a parameter analyzer, and a data acquisition system (DAQ, National Instrument USB‐6363) with the current preamplifier (Model 1211, DL instruments).

### 4.3 Temperature Analysis

Temperature measurements for activation energy calculation were conducted using a vacuum probe station equipped with a temperature‐controlled chuck. The pulse measurements were conducted in the temperature range of 300–323K under vacuum conditions (∼10^−2 ^Torr) to analyze the mechanism of the device.

### 4.4 HOTS Implementation and Simulation

The Poker DVS dataset, which provides spatiotemporal event data of flipped card symbols, was used to implement the HOTS algorithm. The HOTS algorithm consisted of two main steps: online clustering and classification. The online clustering process was used to learn the time surface prototypes. Each time surface was generated over a 7 × 7 pixel spatial window centered on the event location using an exponential decaying kernel. A double exponential decay model was applied to generate the time surfaces to emphasize recent activity while preserving temporal context, with each kernel parameter extracted from device experimental data. The effect of kernel device variations on the performance of the HOTS was studied, and the variations were modeled based on the Gaussian distribution. For the simulation of the conventional HOTS algorithm, a single exponential decaying kernel with an incremental time constant along the layer depth was used to generate the time surfaces [44]. The resulting time surfaces were incrementally clustered using an online learning algorithm, where each surface was assigned to the closest prototype based on similarity, as defined by Equation (2), and the prototypes were updated accordingly [49]:

(2) $$\begin{array}{ccc} & & C_{k}\;\leftarrow \;C_{k}+\alpha \beta \left(S_{i\;}-\;C_{k}\right),\;\;\beta =<C_{k},S_{i}>=\frac{C_{k}\cdot S_{i}}{∥C_{k}∥∥S_{i}∥},\\ & & \alpha =\frac{0.01}{1+\frac{p_{k}}{20000}} \end{array}$$

Here, *S_i_* denotes the time‐surface corresponding to each input event, *C_k_* is the time‐surface prototype most similar to *S_i_*, and *p_k_* represents the number of time surfaces previously assigned to *C_k_*. For classification, the Multi‐Logistic Regression (MLR) classifier was used. The classification process was conducted based on the stream of prototype activations from the clustering results, and the model was optimized using the Adam optimizer.

## Author Contributions

Y. C. and S. C. conceived this work. Y. C., D.‐w. K., S.‐O. P., and T. J. designed and fabricated the device. Y. C. conducted electrical measurements. Y. C., D.‐w. K., and J. B. analyzed the mechanisms of the device. Y. C., D. K., and J. L. designed the device model, performed classification algorithm simulations, and analyzed the results. Y. C. and S. C. prepared the manuscript. S. C. directed the project. All authors contributed to the discussion and analysis of the results regarding the manuscript.

## Conflicts of Interest

The authors declare no conflicts of interest.

## Supporting information




**Supporting File**: advs75594‐sup‐0001‐SuppMat.docx.

## Data Availability

The data that support the findings within this study are available from the corresponding author upon reasonable request.
