# Supplementary material for: Tunable Dynamics via Dual‐Ion Modulation for Event‐based Data Processing Using a Highly Uniform and Self‐Rectifying Memristor Array
Source: Adv Sci (Weinh). 2026 May 10;13(43):e75594. doi: 10.1002/advs.75594 (PMC13336106; doi:10.1002/advs.75594)
Supplement: Supplementary file 1 — Supporting File: advs75594‐sup‐0001‐SuppMat.docx. [file ADVS-13-e75594-s001.docx]

Supporting Information

Tunable Dynamics via Dual-Ion Modulation for Event-based Data Processing Using a Highly Uniform and Self-Rectifying Memristor Array

Yoonho Cho, Dawon Kim, Jeonghong Lee, Dae-won Kim, See-On Park, Jongmin Bae, Taehwan Jang, and Shinhyun Choi*

Y. Cho, D. Kim, J. Bae, S. Choi

School of Electrical Engineering, Korea Advanced Institute of Science and Technology (KAIST), Daejeon 34141, Republic of Korea

E-mail: shinhyun@kaist.ac.kr

J. Lee, D.-w. Kim, T. Jang

Graduate School of Semiconductor Technology, Korea Advanced Institute of Science and Technology (KAIST), Daejeon 34141, Republic of Korea

S.-O. Park

Information and Electronics Research Institute, Korea Advanced Institute of Science and Technology (KAIST), Daejeon 34141, Republic of Korea

Tables of contents

- Supplementary Figures S1-S19
- Supplementary References

Supplementary Figures


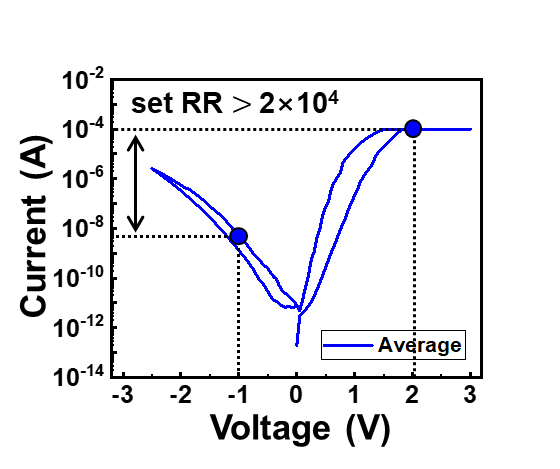


**Figure S1. The set rectifying ratio of the memristor with Ag nanoclusters is obtained as the ratio of the current at positive set voltage to that of negative half set voltage based on the half voltage scheme.**


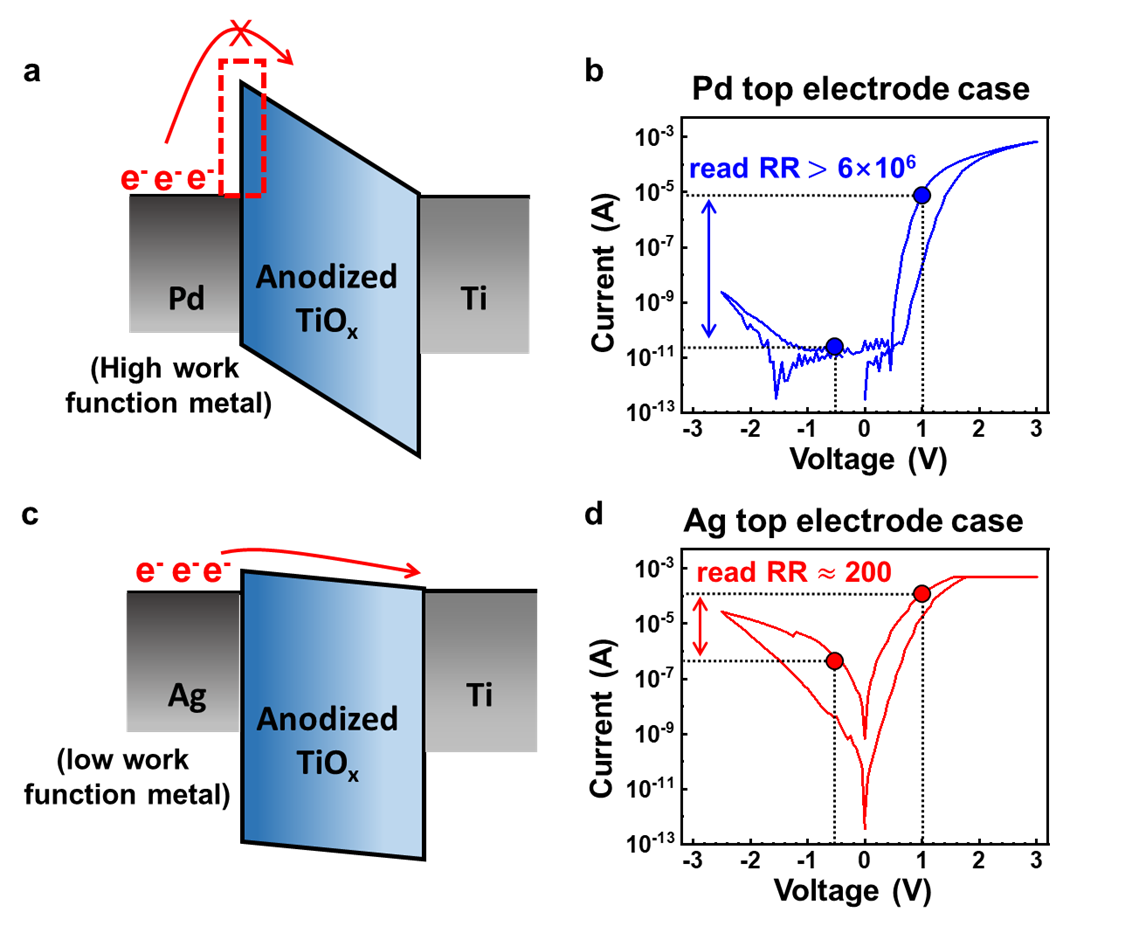


**Figure S2**. **Schematic energy band diagram and the corresponding *I*-*V* curve of the memristor illustrating the effect of the work function of the top electrodes on the read RR.** a) High work function metal Pd (φ = 5.12 eV^[1]^) electrode case, b) Corresponding *I*-*V* curve and read RR of the device, c) Ag (φ = 4.26 eV^[2]^) top electrode case, d) Corresponding *I*-*V* curve and read RR of the device.


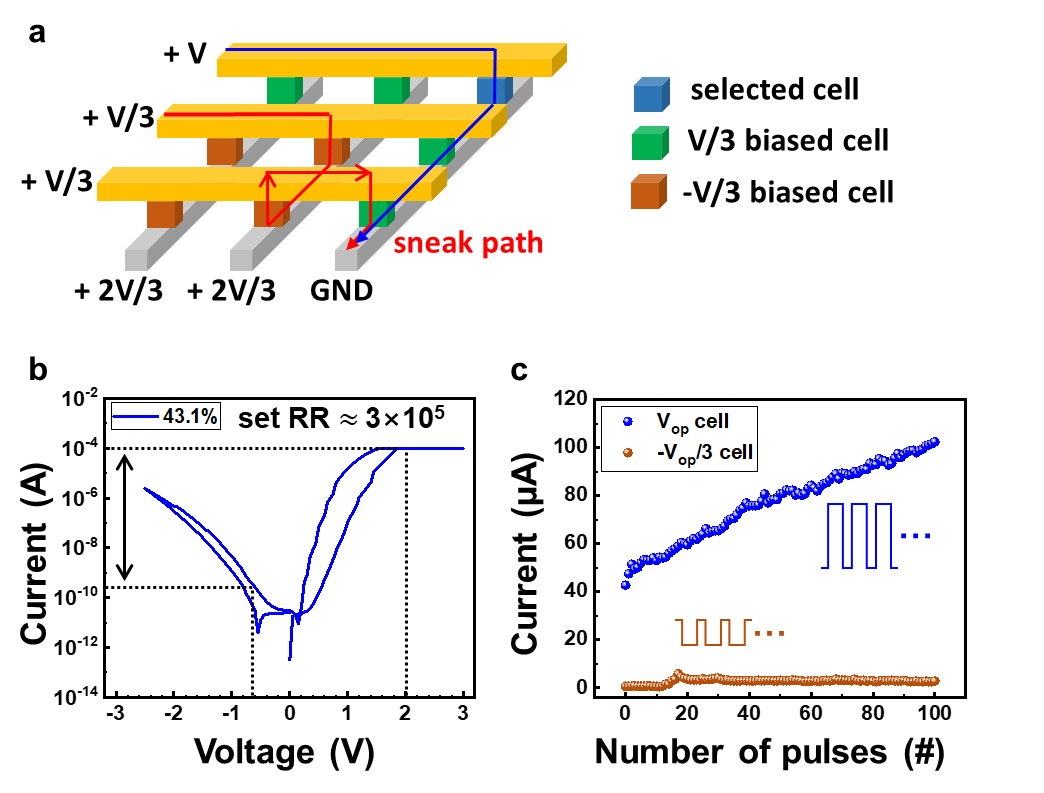


**Figure S3. Effect of the self-rectifying behavior of the memristor with Ag nanoclusters in the memristor operation.** a) Schematic illustrating the potential sneak current paths in the array based on a one-third biasing scheme, b) High set RR calculated in the *I*-*V* curve according to a one-third biasing scheme, c) Pulse response of the memristor of the selected cell (V_set_ applied) and -V_set_/3 biased cell in the array under a one-third biasing scheme.


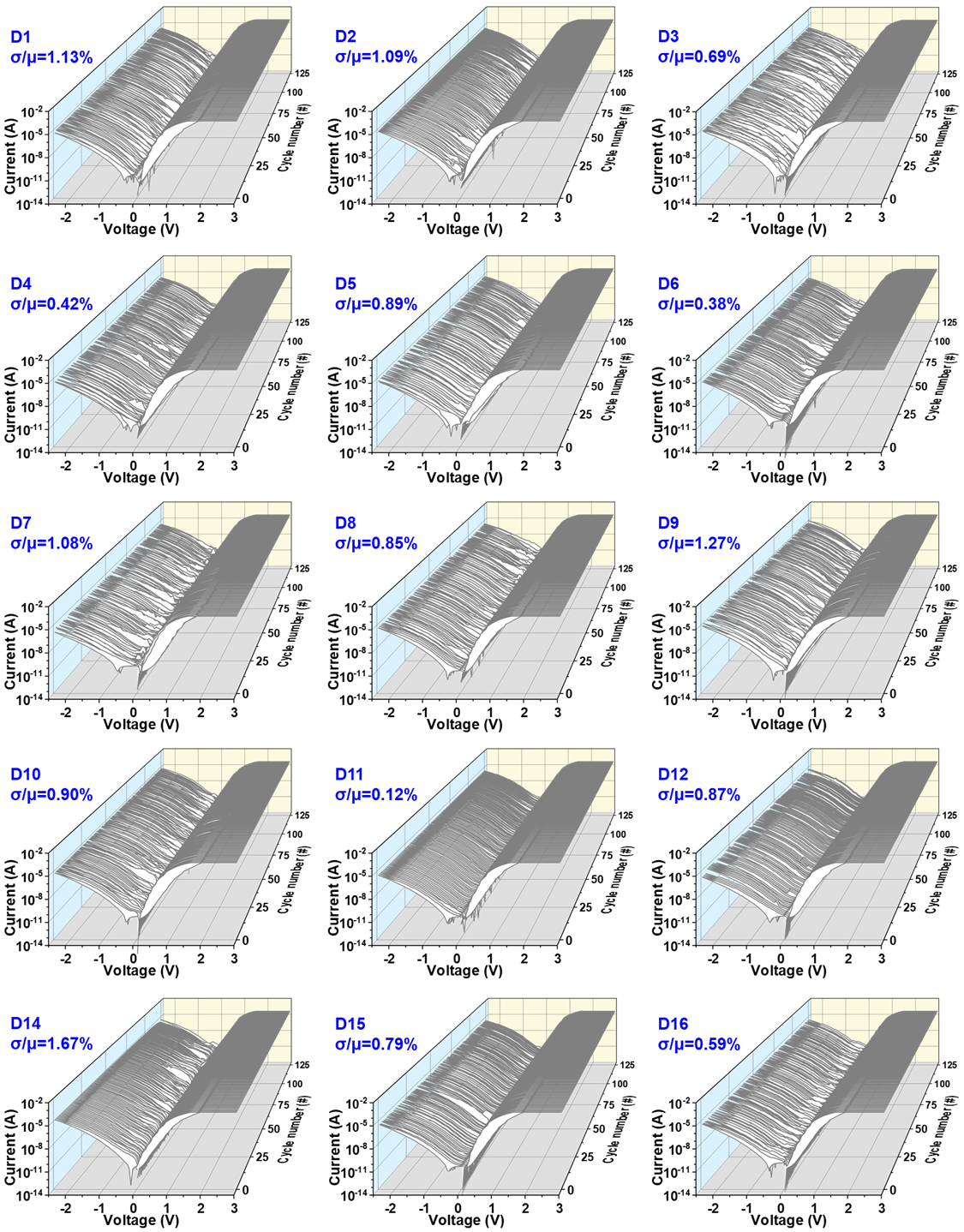


**Figure S4.** **Analysis of the temporal variation by applying consecutive 125 DC cycles in 15 devices within the array.**


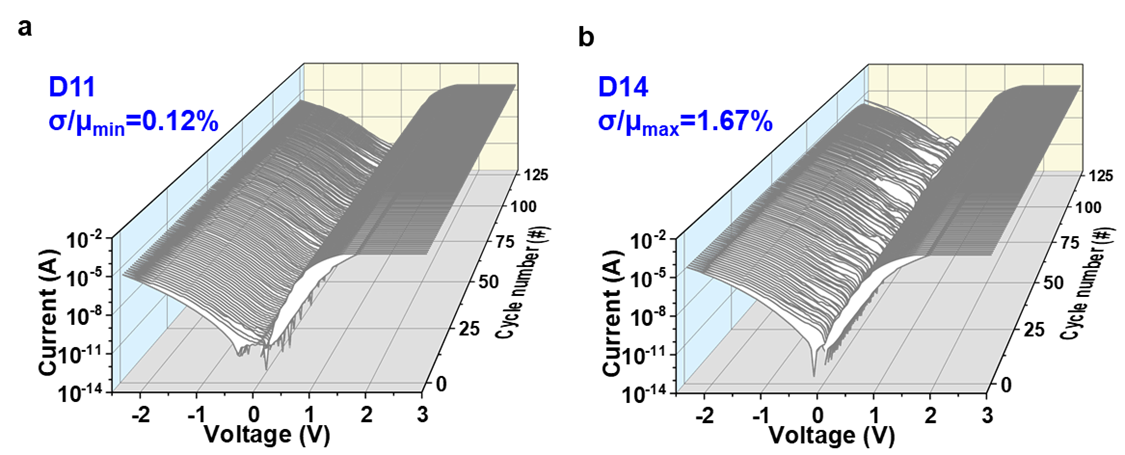


**Figure S5. The results of consecutive 125 DC cycles showing the minimum and maximum temporal variations.**


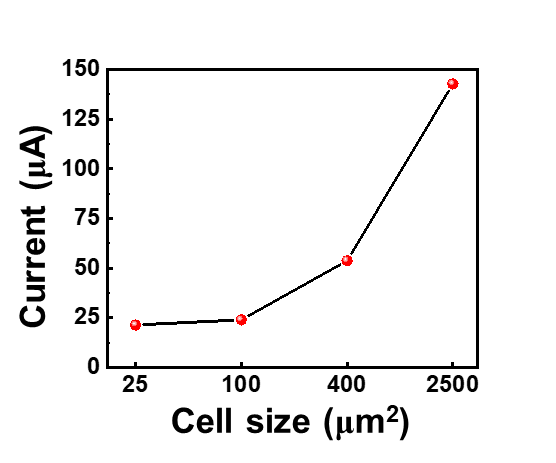


**Figure S6. Size dependency of the memristor with Ag nanoclusters.** The currents of four different sizes (5×5, 10×10, 20×20, and 50×50 (μm^2^)) were measured at 1 V during the DC sweep.


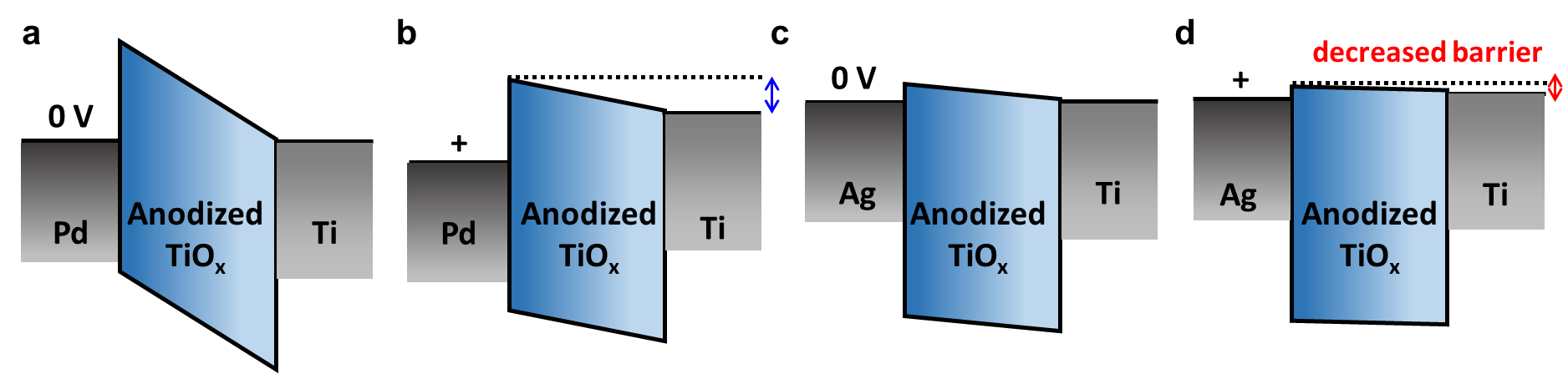


**Figure S7.** **Schematic energy band diagram illustrating the effect of the work function of the top electrodes on the current level.** High work function metal Pd electrode case a) under zero bias, b) small positive bias, c) Low work function metal Ag electrode case c) under zero bias, d) small positive bias.

**
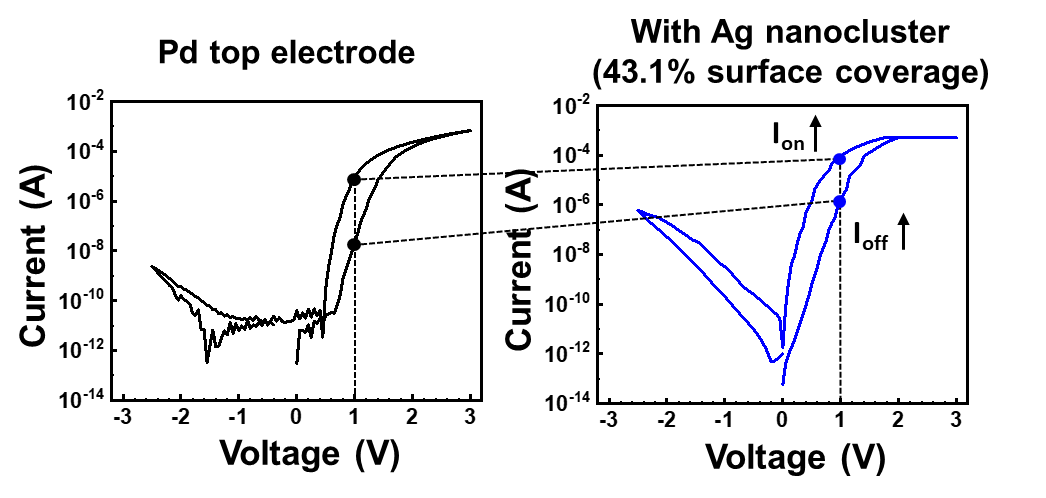
**

**Figure S8.** **The comparison of the *I*-*V* curve between the meristor without and with Ag nanoclusters.** An increase in the current of both HRS and LRS is observed when Ag nanoclusters are inserted due to the combined effect of Pd and Ag on the conduction mechanism.


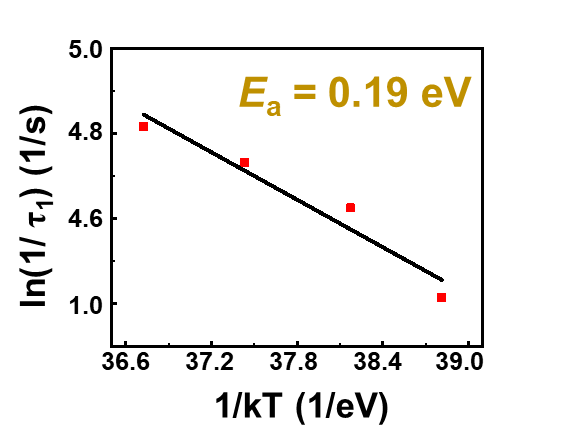


**Figure S9. Activation energy calculated from the time constants of the first exponential term at various temperatures in the memristor with Ag nanoclusters.**


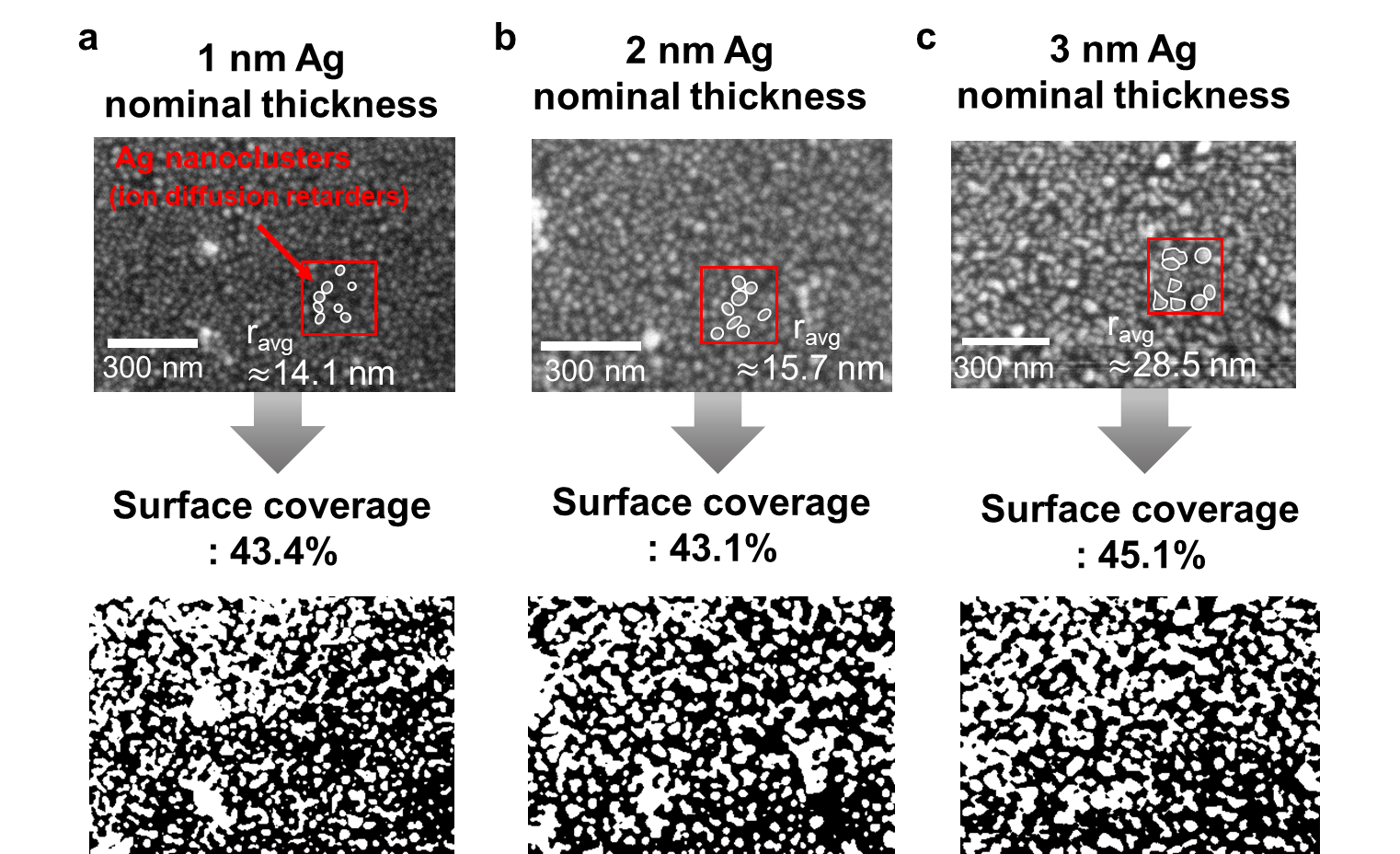


**Figure S10. Scanning electron microscope (SEM) image of the memristor with different amounts of Ag nanoclusters.** a) 43.4% Ag surface coverage, b) 43.1% Ag surface coverage, c) 45.1% Ag surface coverage.


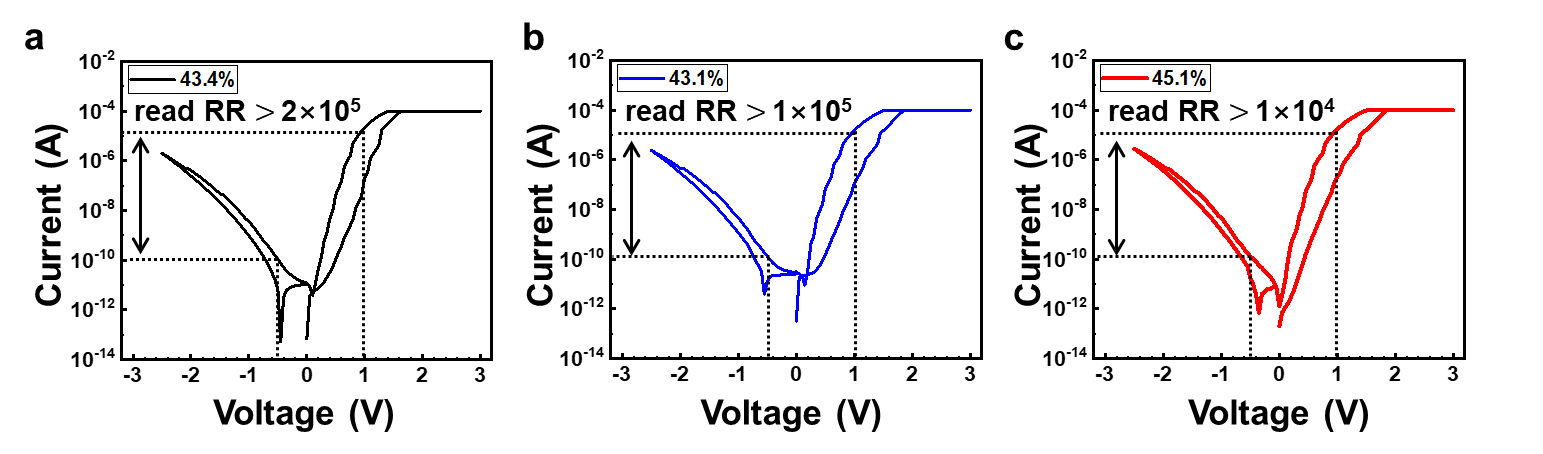
 **Figure S11. *I*-*V* characteristics of the memristor with different amounts of Ag nanoclusters.** Ag nominal thickness of a) 1 nm (43.4% surface coverage), b) 2 nm (43.1% surface coverage), and c) 3 nm (45.1% surface coverage).


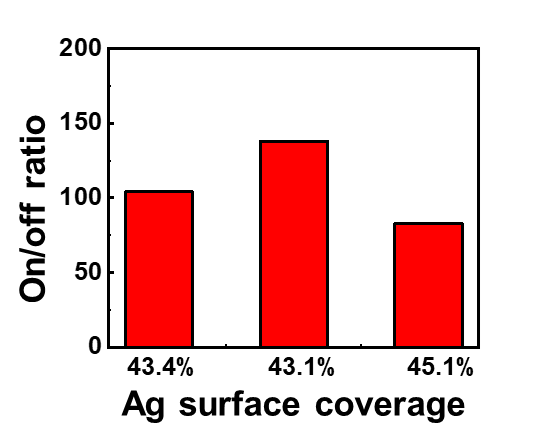


**Figure S12. Histogram showing the on/off ratio of the memristor with different amounts of Ag nanoclusters.**


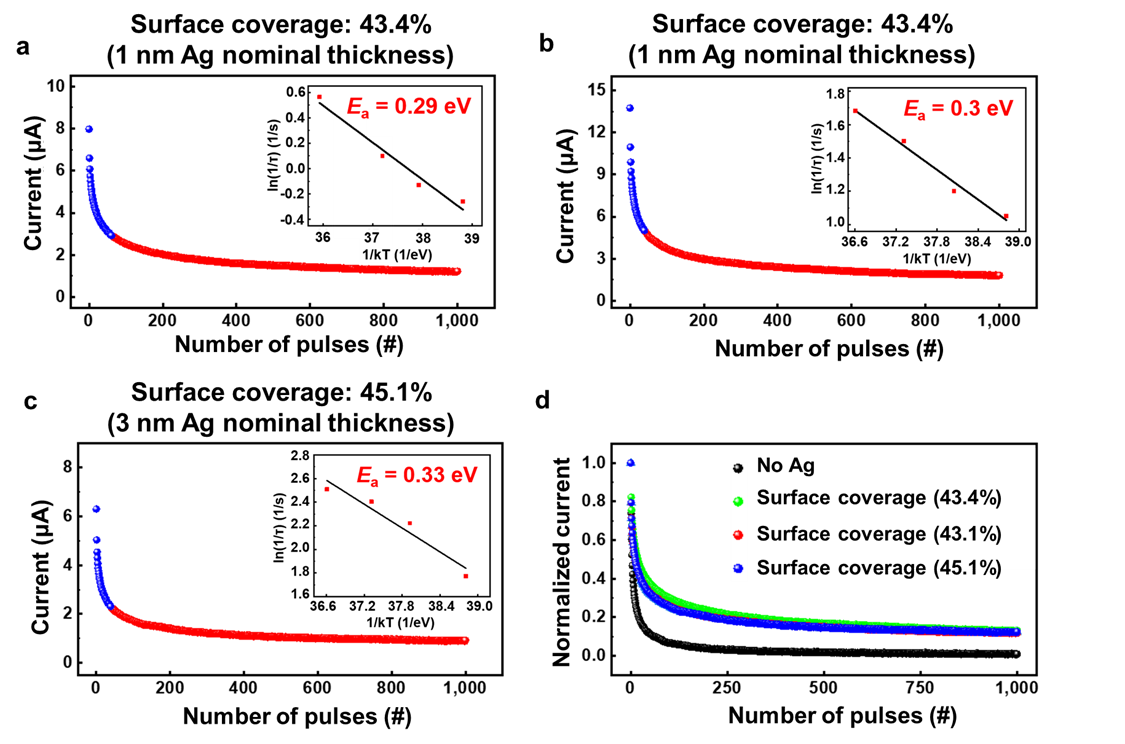


**Figure S13. Decaying characteristics of the memristor with different amounts of Ag nanoclusters.** a) with 43.4% Ag nanocluster surface coverage, b) with 43.1% Ag nanocluster surface coverage, c) with 45.1% Ag nanocluster surface coverage, d) normalized decaying characteristics of memristor with various amounts of Ag nanoclusters.


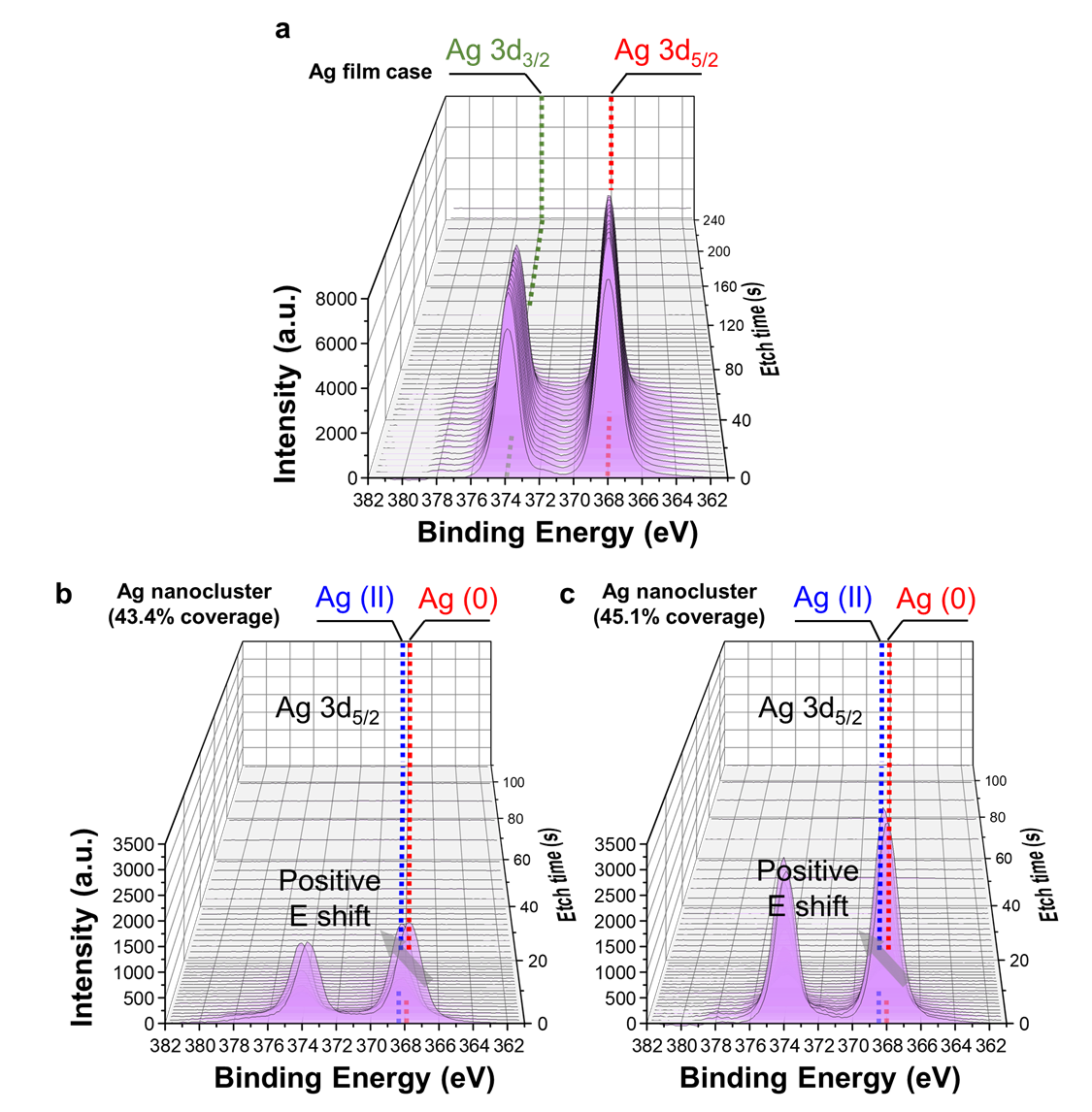


**Figure S14**. **Ag 3d orbitals X-ray** **photoelectron spectroscopy (XPS) depth profile results of the interface-type memristor with various Ag content.** a) Ag film case. b,c) Ag nanoclusters with b) 43.4% surface coverage, c) 45.1% surface coverage. Positive peak shifts of Ag 3d5/2 spectra are observed for the case of 43.4% and 45.1% surface coverage as the distance from the surface increases.


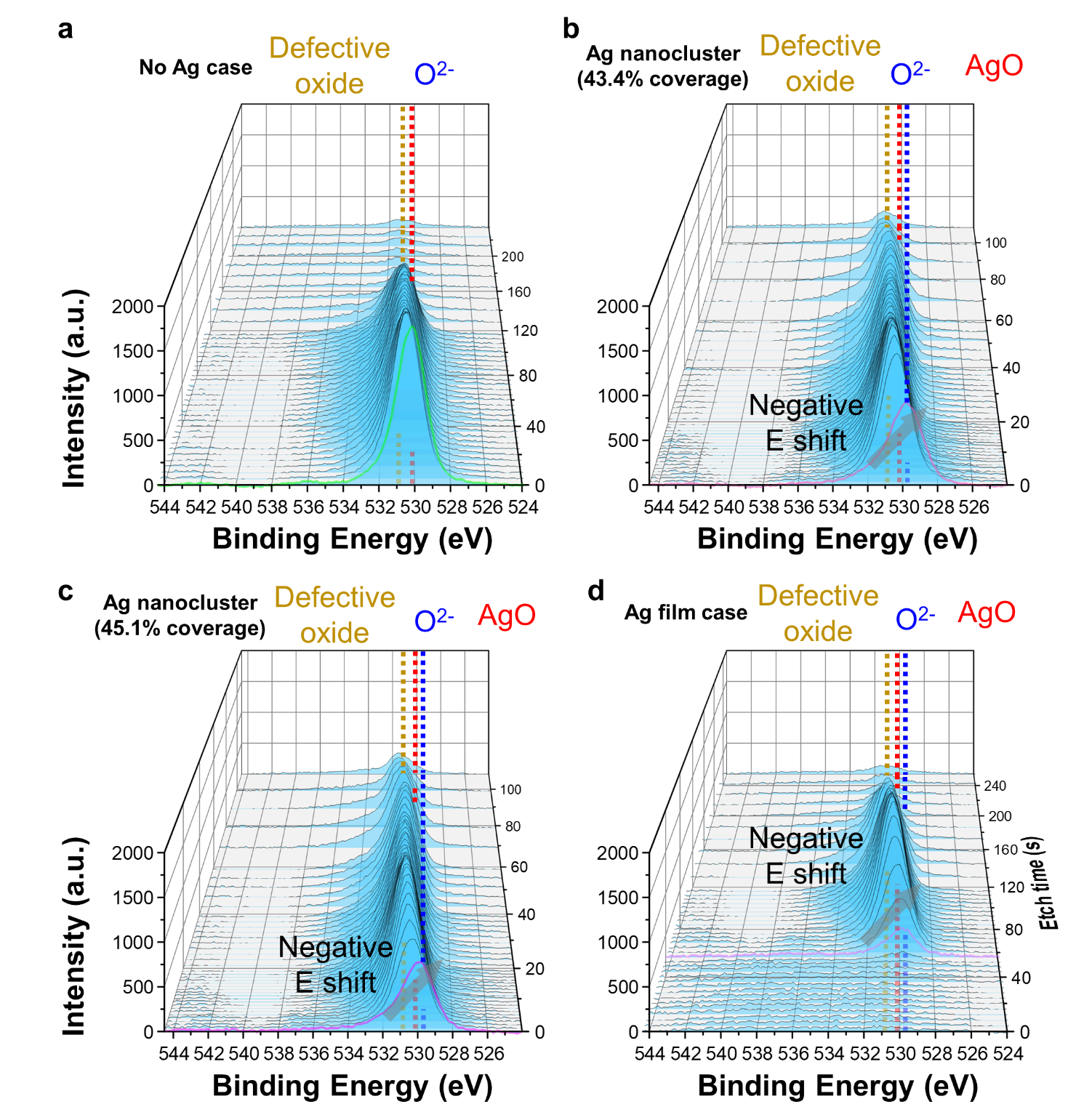


**Figure S15**. **O 1s XPS depth profile results of the interface-type memristor with various Ag content.** a) No Ag case, b,c) Ag nanoclusters with b) 43.4% surface coverage, and c) 45.1% surface coverage, and d) Ag film case. Negative peak shifts in the device with the addition of Ag are observed, regardless of Ag content.


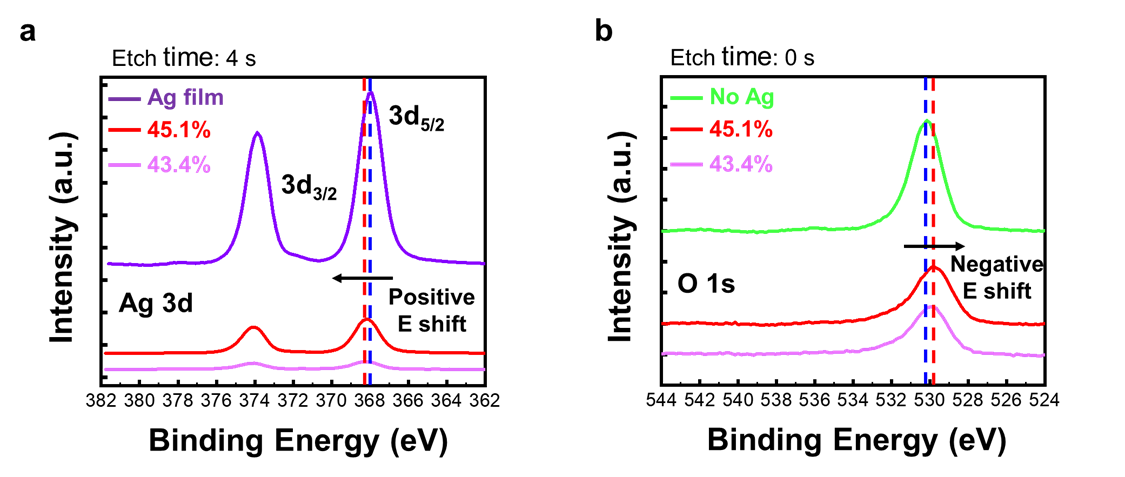


**Figure S16**. **Ag 3d and O 1s XPS spectra of the memristor with various Ag content.** a) Ag 3d XPS spectra of the interface-type memristor with various Ag contents (43.4% surface coverage (pink curve), 45.1% surface coverage (red curve), and Ag film (purple curve)) at 4 s etch time. Negative peak shifts are observed with the addition of Ag nanoclusters, b) O 1s XPS spectra of the interface-type memristor with various Ag contents (without Ag (green curve), 43.4% surface coverage (pink curve), and 45.1% surface coverage (red curve)) at the surface (0 s etch time). Negative peak shifts are observed with the addition of Ag nanoclusters.

**
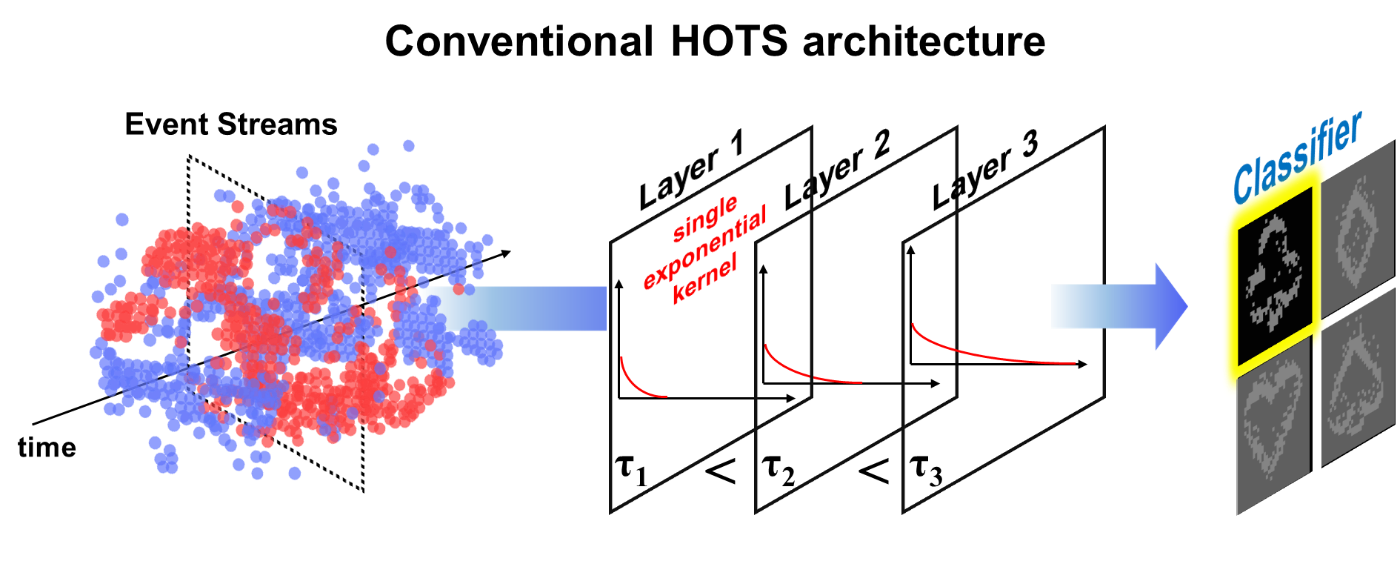
Figure S17.** **Schematics of processing event-based pattern recognition in the conventional hierarchy of the event-based time surface (HOTS) algorithm.^[3]^** Time surfaces are generated using a single exponential decaying kernel. As the layer deepens, the time constants of the layers increase to integrate multiple time scales of the inputs.


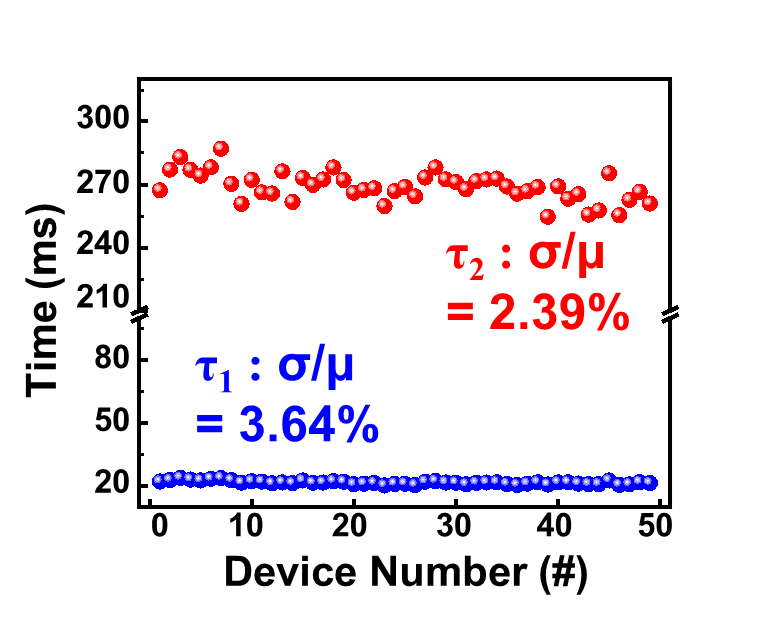


**Figure S18.** **Highly uniform characteristics of the hardware-implemented exponential decaying kernel using a 7×7 array of interface-type memristors with Ag nanoclusters**. The variation for the time constant in the first exponential term (τ_1_) and the second exponential term (τ_2_) was 3.64% and 2.39%, respectively, across all 49 measured devices.


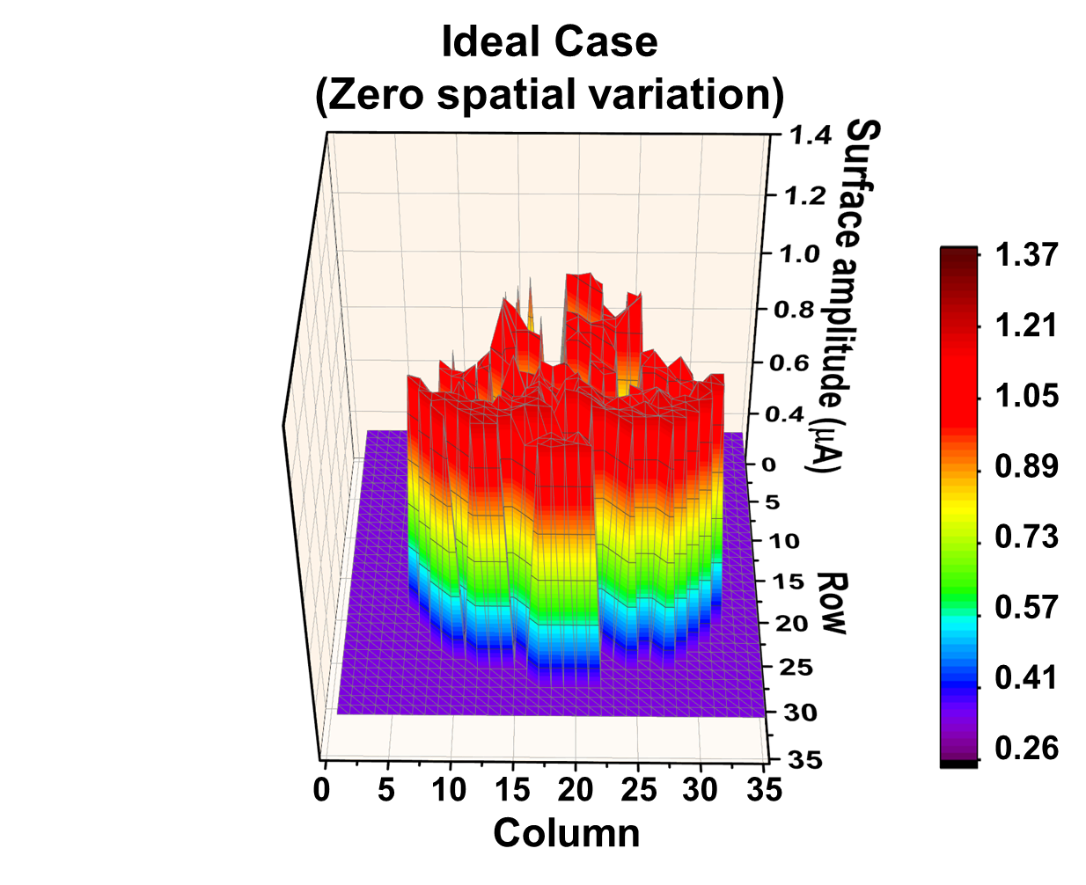


**Figure S19. The result of the time surface generated by using an ideal exponential decaying kernel with zero spatial variation, where all kernels show identical decaying properties.**

**Supplementary References**

[1] S. Kim, J. Zhou, W. D. Lu, *IEEE Trans. Electron Device* **2014**, 61, 2820.

[2] C. Vallée, P. Gonon, C. Jorel, F. El Kamel, *Appl. Phys. Lett.* **2010**, 96. 233504

[3] X. Lagorce, G. Orchard, F. Galluppi, B. E. Shi, R. B. Benosman, *IEEE Trans. Pattern Anal. Mach. Intell.* **2017**, 39, 1346.
